# Supplementary material for: Structures of highly flexible intracellular domain of human α7 nicotinic acetylcholine receptor
Source: Nat Commun. 2022 Feb 10;13:793. doi: 10.1038/s41467-022-28400-x (PMC8831596; doi:10.1038/s41467-022-28400-x)
Supplement: Supplementary file 1 — Supplementary Information [file 41467_2022_28400_MOESM1_ESM.pdf]

Supplementary Information

Structures of Highly Flexible Intracellular Domain of Human α7 Nicotinic Acetylcholine Receptor

Vasyl Bondarenko[1,9], Marta M. Wells[1,9], Qiang Chen[1], Tommy S. Tillman[1], Kevin Singewald[2], Matthew J. Lawless[2], Joel Caporoso[1], Nicole Brandon[1], Jonathan A. Coleman[3], Sunil Saxena[2], Erik Lindahl[4,5], Yan Xu[1,3,6,7], Pei Tang[1,6,8*]

[1]Depatment of Anesthesiology and Perioperative Medicine, University of Pittsburgh, Pittsburgh, PA 15260, USA
[2]Department of Chemistry, University of Pittsburgh, Pittsburgh, PA 15260, USA
[3]Department of Structural Biology, University of Pittsburgh, Pittsburgh, PA 15260, USA
[4]Department of Biochemistry and Biophysics, Science for Life Laboratory, Stockholm University, Solna, Sweden
[5]Department of Applied Physics, Swedish e-Science Research Center, KTH Royal Institute of Technology, Solna, Sweden
[6]Department of Pharmacology and Chemical Biology, University of Pittsburgh, Pittsburgh, PA 15260, USA
[7]Department of Physics and Astronomy, University of Pittsburgh, Pittsburgh, PA 15260, USA;
[8]Department of Computational and Systems Biology, University of Pittsburgh, Pittsburgh, PA 15260, USA
[9]Contributed equally to this study
*Correspondence and requests for materials should be addressed to P.T. (ptang@pitt.edu)

Supplementary Figures 1-15

Supplementary Tables 1-5

```
                          209            TM1                         TM2
α7nAChR(TMD+ICD)NMR SNAEEELYYGLNLLIPCVLISALALLVFLLPADSGEKISLGITVLLSLTVFMLLVAEIMP 262
α7nAChR(TMD+ICD)    ------LYYGLNLLIPCVLISALALLVFLLPADSGEKISLGITVLLSLTVFMLLVAEIMP

                                   TM3                                MX
α7nAChR(TMD+ICD)NMR STSDSSPSIAQYFASTMIIVGLSVVVTVIVLQYHHHDPDGGKMPKWTRVILLNWCAWFLR 322
α7nAChR(TMD+ICD)    ATSDSVPLIAQYFASTMIIVGLSVVVTVIVLQYHHHDPDGGKMPKWTRVILLNWCAWFLR

                        h1      h2                      h3
α7nAChR(TMD+ICD)NMR MKRPGEDKVRPACQHKQRRCSLASVEMSAVAPPPASNGNLLYIGFRGLDGVHCVPTPDSG 382
α7nAChR(TMD+ICD)    MKRPGEDKVRPACQHKQRRCSLASVEMSAVAPPPASNGNLLYIGFRGLDGVHCVPTPDSG

                                                                  MA
α7nAChR(TMD+ICD)NMR VVCGRMACSPTHDEHLLHGGQPPEGDPDLAKILEEVRYIANRFRCQDESEAVCSEWKFAA 442
α7nAChR(TMD+ICD)    VVCGRMACSPTHDEHLLHGGQPPEGDPDLAKILEEVRYIANRFRCQDESEAVCSEWKFAA

                                 TM4
α7nAChR(TMD+ICD)NMR CVVDRLCLMAFSVFTIICTIGILMSAPNFVEE 474
α7nAChR(TMD+ICD)    CVVDRLCLMAFSVFTIICTIGILMSAPNFVEAVSKDFA 480
```

Supplementary Fig. 1. **Sequence comparisons of the α7nAChR TMD+ICD construct used for NMR with the original sequence**. The residues highlighted in the orange color were introduced to the NMR construct to improve the protein solubility and stability[1]. Residues L209-V472 were used in structure calculations. The sequence alignment was obtained using Clustal Omega 1.2.4 at Uniprot (https://www.uniprot.org/align/).

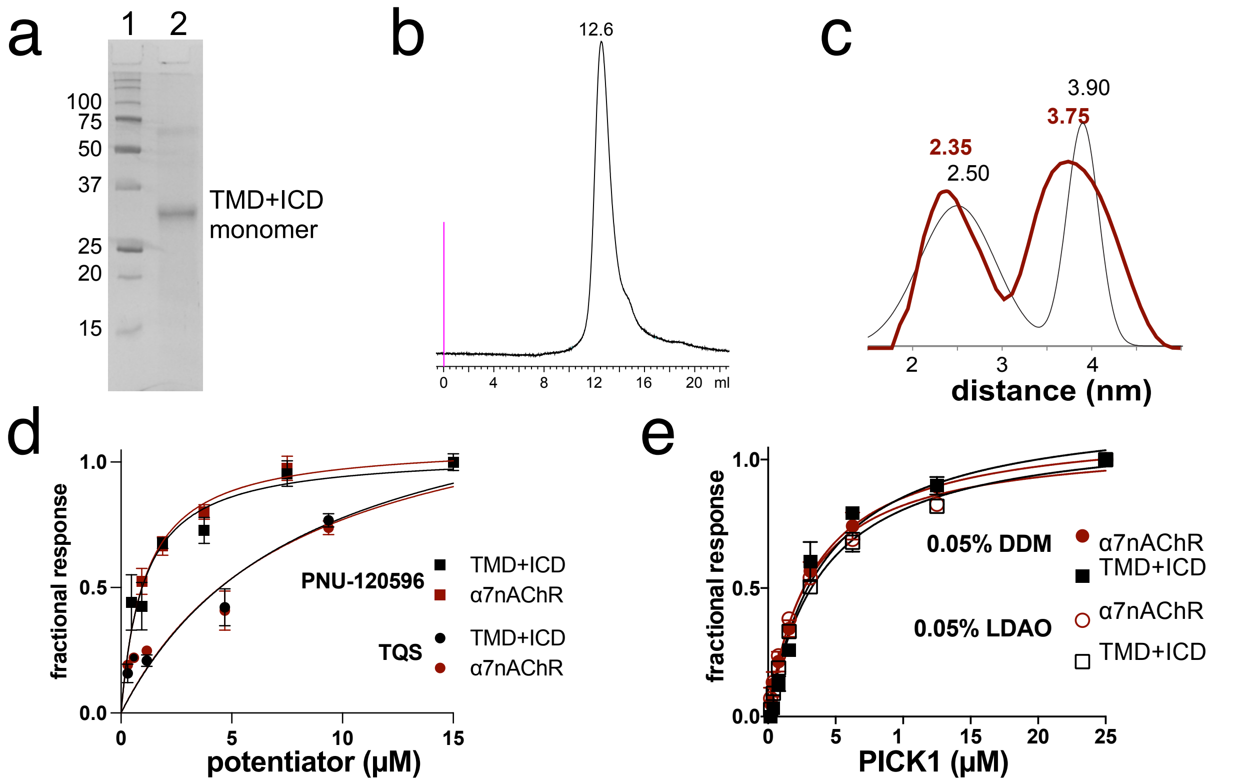

Supplementary Fig. 2. **The pentameric α7nAChR TMD+ICD used for NMR structural studies.** (**a**) 15% SDS PAGE gel showing molecular weight markers (lane 1 in kilodaltons) and purified TMD+ICD (lane 2). Protein quality was assessed for each purification. The identity of the monomer band was confirmed to be the TMD+ICD by MS/MS after tryptic digestion at the UPCI Cancer Biomarkers Facility. (**b**) Size exclusion chromatogram was collected using a Superdex 200 10/300 Increase GL column equilibrated with a buffer containing 10 mM sodium acetate at pH 4.8, 100 mM NaCl, and 0.05% LDAO. The injection point at 0 ml is shown by a vertical pink line. The elution peak at 12.6 ml is consistent with the TMD+ICD pentamer molecular weight. (**c**) The TMD+ICD construct used for NMR forms pentamers that show a typical non-adjacent/adjacent distance ratio (~1.6)[2] in DEER ESR[3] experiments. Data were collected from the TMD+ICD C219 (red) in LDAO micelles and full-length α7nAChR C435 (black) in liposomes. Both constructs formed pentamers. (**d**) PNU-120596 and TQS, positive allosteric modulators (PAMs) of α7nAChR, are known to bind the TMD. In surface plasmon resonance (SPR) measurements, each of the PAMs shows similar binding affinity to the TMD+ICD (black: K_D = 1.0±0.2 μM for PNU-120596; 8.2±2.2 μM for TQS) and the full-length α7nAChR (red: K_D = 1.1±0.1 μM for PNU-120596; 7.8±3.2 μM for TQS), suggesting that the TMD+ICD retains the structural integrity of the full length α7nAChR. Both the full-length α7nAChR and TMD+ICD were solubilized in 0.05% LDAO. Note that the measured binding affinities are close to previously reported EC_{50} values (0.2 ± 0.06 μM for PNU-120596[4]; 6.2 ± 0.6 μM for TQS[5]) measured in different types of cells. (**e**) The intracellular scaffold protein PICK1 PDZ domain binds the TMD+ICD (black: K_D = 4.2±0.6 μM in DDM; K_D = 4.0±0.2 μM in LDAO) and the full length α7nAChR (red: K_D = 3.3±0.2 μM in DDM; K_D =3.1±0.2 μM or in LDAO) with similar affinities in SPR measurements. 0.05% LDAO or 0.05% DDM makes no significant difference to the binding results. For SPR measurements in **d** and **e**, data are presented as mean values +/- SD, n=3 independent experiments. Source data are provided as a Source Data file.

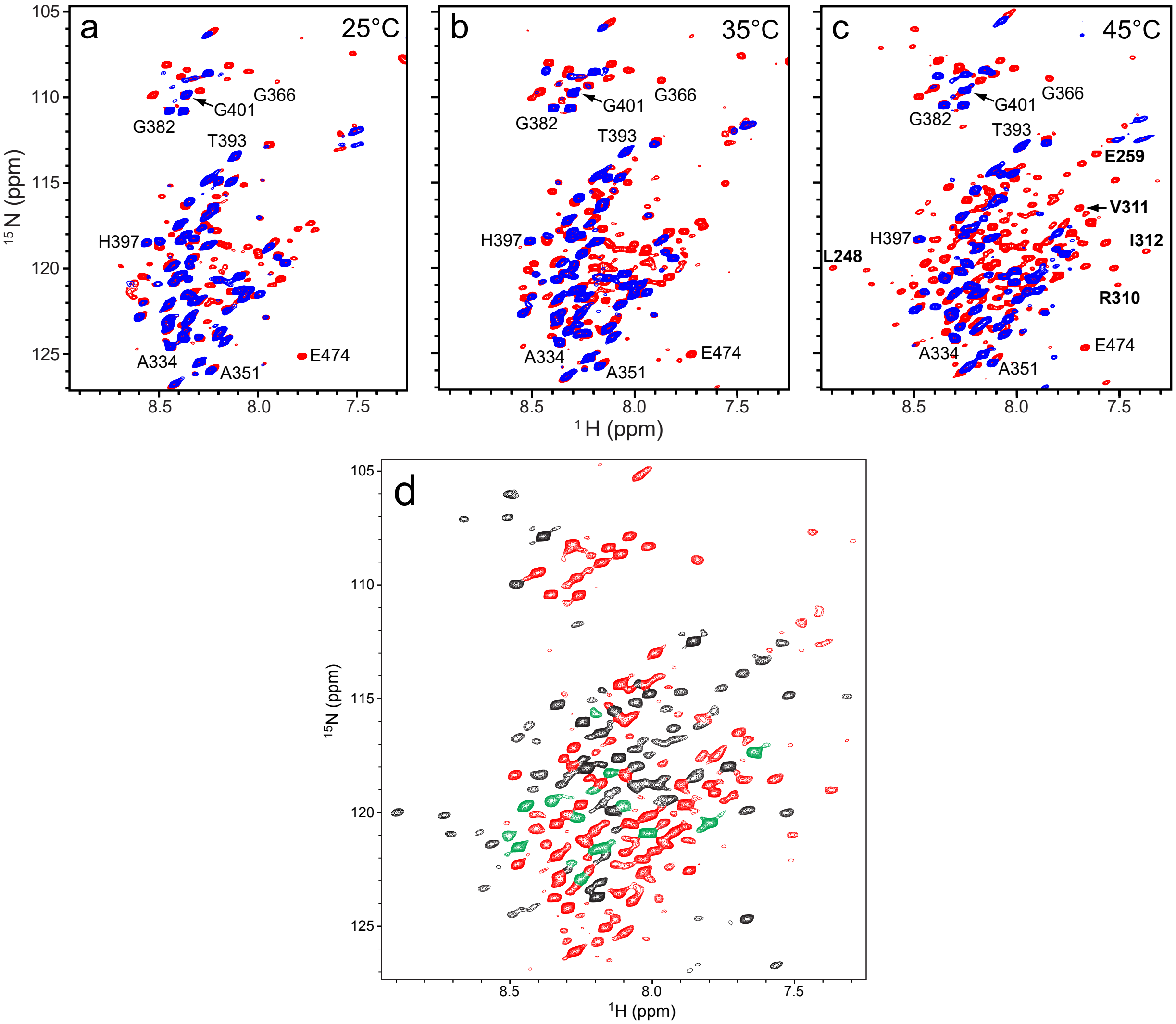

Supplementary Fig. 3. **Comparison of different membrane mimetics and temperatures on the quality of ^{1}H-^{15}N TROSY-HSQC NMR spectra of α7nAChR TMD+ICD.** Overlay of the NMR spectra acquired in LDAO micelles (red) and asolectin lipid nanodiscs (blue) at (**a**) 25ºC, (**b**) 35ºC, and (**c**) 45ºC. A greater number of NMR peaks were observed for samples prepared in LDAO micelles than those prepared in nanodiscs at three different temperatures. Also, there is a clear trend of increasing in number of observed residues when the data collection temperatures raised from 25ºC to 45ºC. For clarity, residues were selectively labeled for different scenarios: residues shown in both micelles and nanodiscs (such as G382, T393 and many others in loop L), residues shown only in micelles at all tested temperatures (such as G366, E474), and residues shown only in micelles and only at 45ºC (such as those residues labeled in bold in **c** that belong to long helices in the TMD or ICD). (**d**) A representative ^{1}H-^{15}N TROSY-HSQC NMR spectrum of α7nAChR TMD+ICD in LDAO micelles collected at 45ºC, where NMR peaks from residues in different domains are color coded: black-the TMD; red- the ICD; green- the overlapped peaks from the ICD with the TMD. Note that the TMD residues have generally weaker intensity except those at the n-terminus and c-terminus. The chemical shift values have been deposited in the Biological Magnetic Resonance Data Bank (BMRB), accession number 30939.

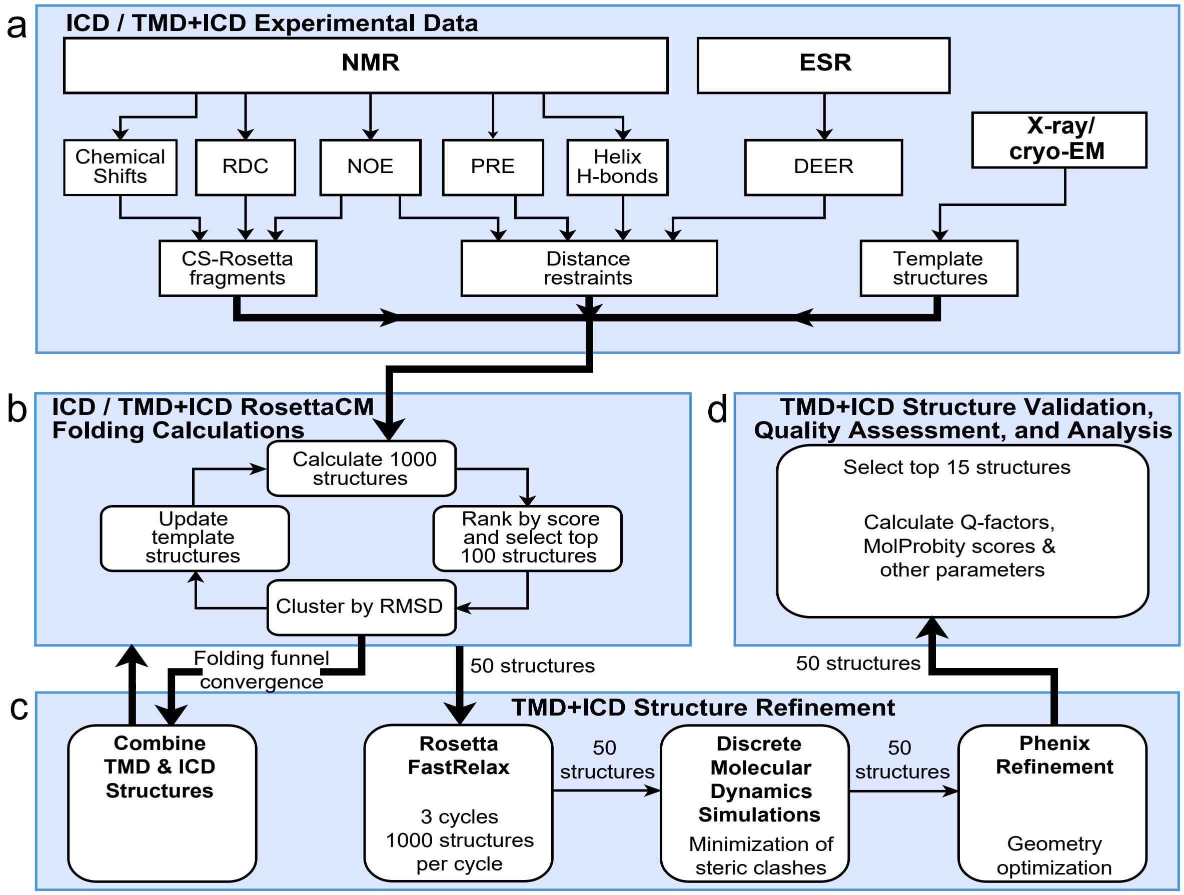

Supplementary Fig. 4. Overview of iterative structure determination procedures. (**a**) Input experimental restraints for structure determinations of the α7nAChR ICD or TMD+ICD. In addition to various NMR restraints (see representative data in Supplementary Figs 5-9) and EPR DEER distance restraints (see representative data in Supplementary Fig 10), the first run of calculations (round 1) in Rosetta also used two template structures, 5-HT$_3$ receptors (PDBID: 6BE1 and 4PIR)[6,7] that were available at the time when the project began. (**b**) Iterative folding calculations with RosettaCM[8]. Folding of the ICD (used residues I291 – C449) converged after 6 rounds and the TMD+ICD structures (residues L209-V472) converged after 16 rounds (see Supplementary Fig. 4e and 4f next page). (**c**) TMD+ ICD structure refinement began with Rosetta FastRelax[9,10,11] that consists of cycles of packing and all-atom minimization and were performed using Open Science Grid[12,13]. The top 50 total-score-ranked structures were selected for discrete MD simulations in Chiron[14] to minimize steric clashes, and were followed by geometry optimization using Phenix[15] (version 1.19). **(d)** Structure evaluation and validation with Q-factor[16,17,18] (analogous to the crystallographic R-factor) as defined by

$$Q = \sqrt{\frac{\sum_i (r_{exp}(i) - r_{calc}(i))^2}{\sum_i r_{\exp}(i)^2}},$$

where r_{exp} is the experimental restraint distance and r_{calc} is the distance observed in the calculated structure. Q-factors were calculated separately for restraints from NMR PRE ($Q^{PRE}{}_{work}$) and EPR DEER (Q^{DEER}). In addition, ~10% of the PRE restraints were randomly selected to be excluded from the structure calculations for validation purposes. Q-factors for these restraints ($Q^{PRE}{}_{free}$) were

calculated based on these restraints. Structures were also evaluated by the MolProbity scores, and other parameters as reported in Supplementary Table 2.

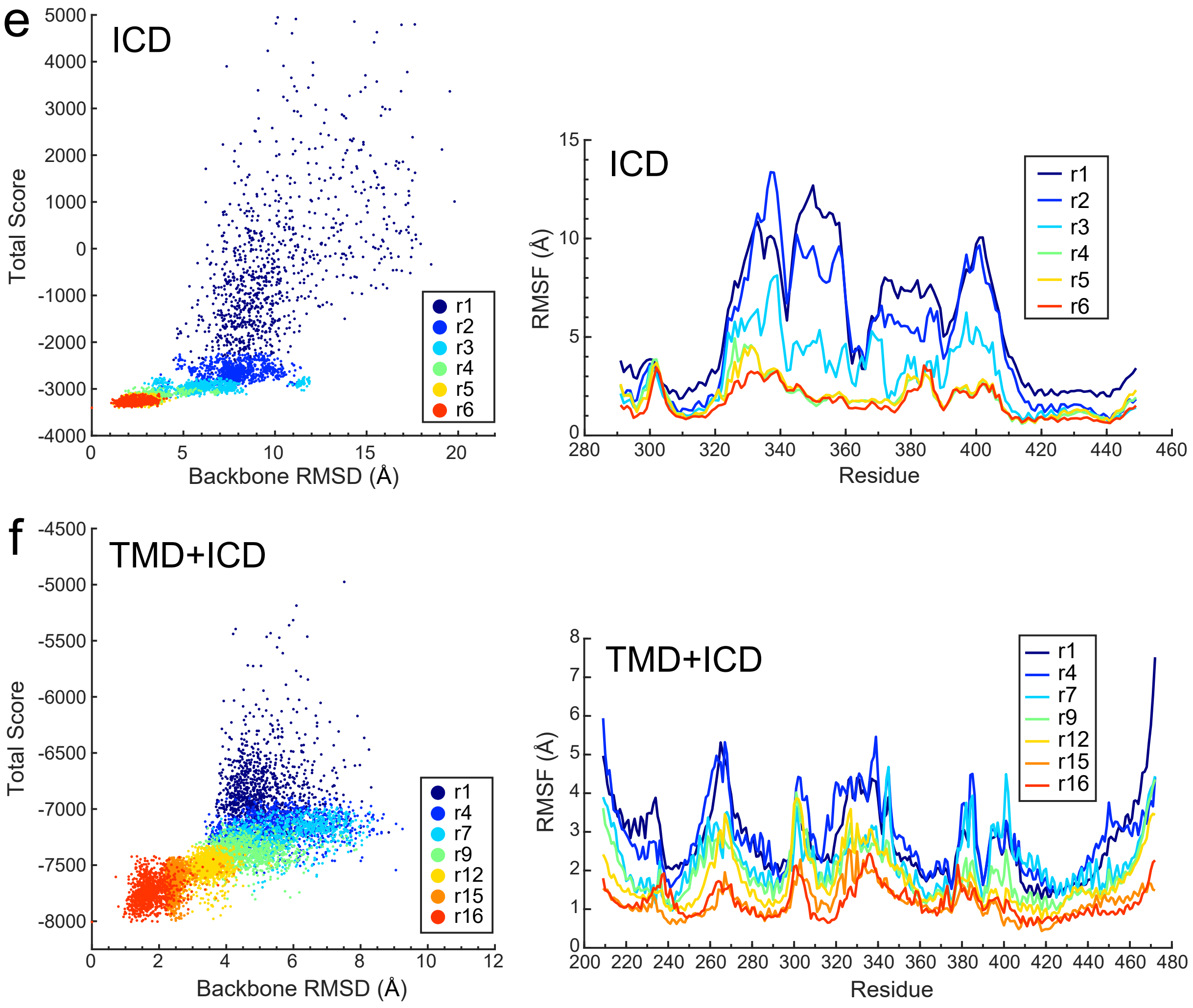

Supplementary Fig. 4. (Continuation from the previous page) **Evolution of structural convergence in iterative Rosetta calculations. (e)** Evolution of ICD structural convergence. (left) Backbone RMSD values of 1000 structures were calculated for each round with respect to the best scoring structure in the final round (r6). Total score includes Rosetta energy and experimental restraint score. (right) Cα RMSF values were calculated for the 100 top-scored structures in each cycle. (**f**) Evolution of TMD+ICD structural convergence. (left) Backbone RMSD values of 1000 structures vs. their total scores and (right) Cα RMSF values of the 100 top-scored TMD+ICD structures in each round vs. individual residues were calculated. For clarity, only representative rounds (as marked) are shown. Source data are provided as a Source Data file.

Representative experimental restraints used in structure determination (Supplementary Fig. 5 - Fig. 10)

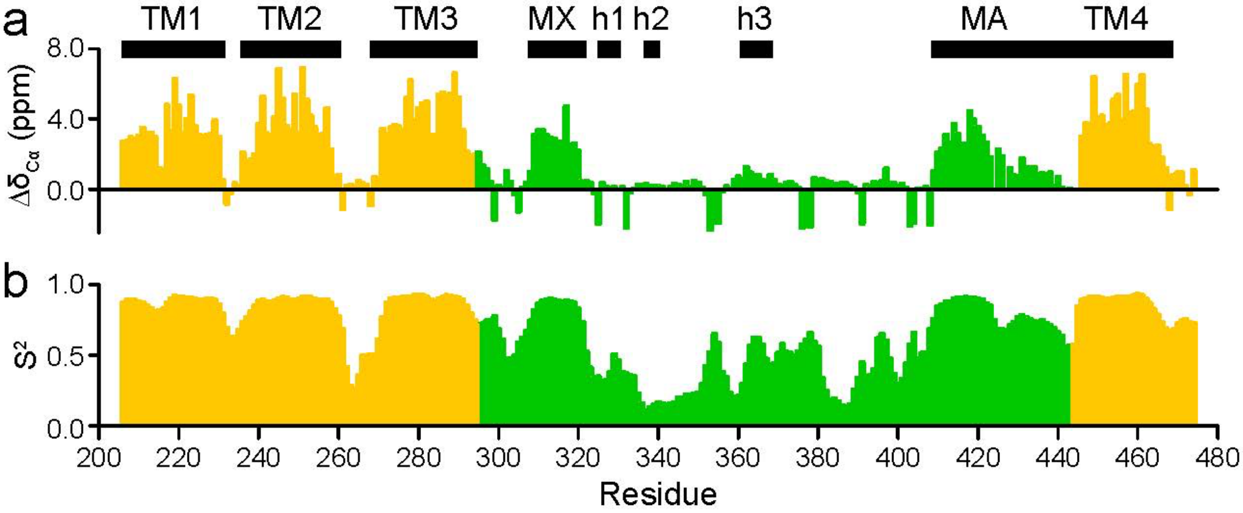

Supplementary Fig. 5. **NMR secondary structure determination**. (**a**) Cα chemical shift index determined by the difference ($\Delta\delta_{C\alpha}$) between the observed Cα chemical shift and that of the corresponding amino acid in a random coil conformation. $\Delta\delta_{C\alpha}$ values greater than zero are indicative of helical structure and smaller than zero are indicative of β-strand[19]. Chemical shifts were used for generating 3- and 9-residues fragments using CS-Rosetta[20] and for secondary structure determinations using TALOS+[21]. (**b**) Order parameters (S^2) derived from Cα, Cβ, CO, N, and HN chemical shifts for each residue using TALOS+[21]. S^2 is a general measure of flexibility (1 = rigid). TMD and ICD residues are shown in gold and green, respectively. Four TM helices, MA and MX helices, and additional short α-helices (h1, h3) and 3-10 helix (h2) are marked with horizontal bars. Source data are provided as a Source data file.

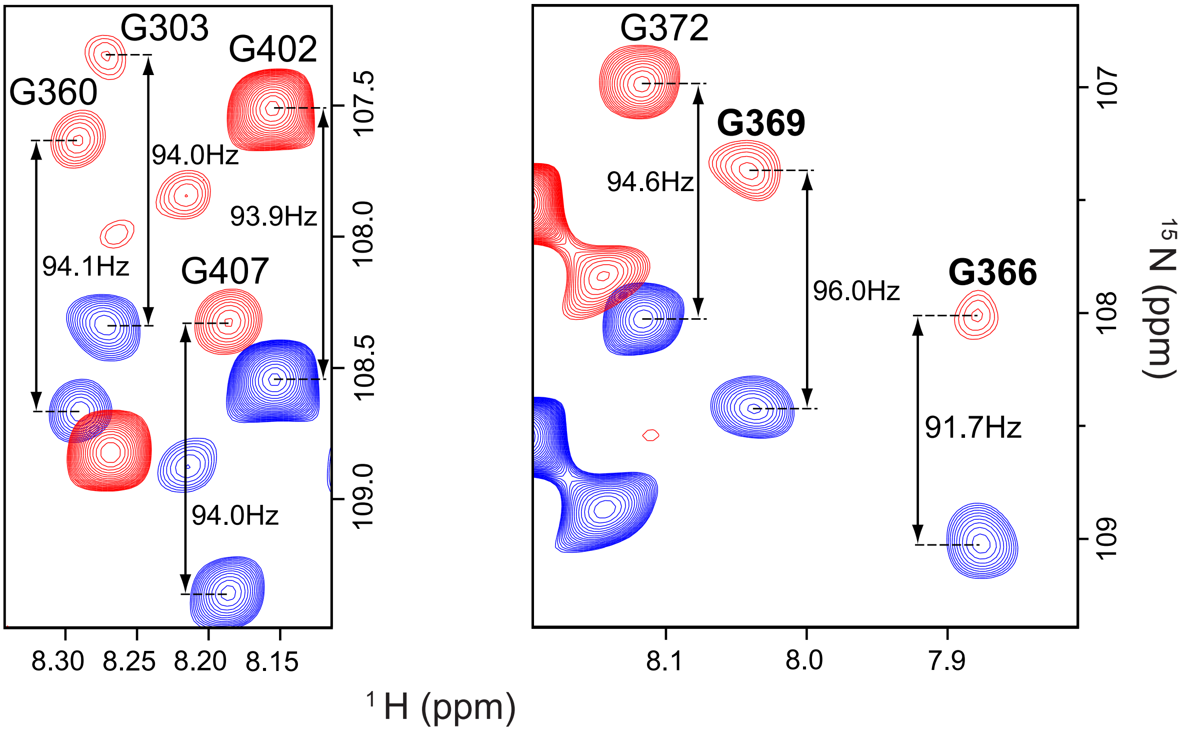

Supplementary Fig. 6. Residual Dipole Couplings (RDCs) revealed by ^{1}H-^{15}N in-phase/anti-phase (IPAP) TROSY NMR experiments. Representative zoom-in regions from ^{1}H-^{15}N IPAP spectra of α7nAChR TMD+ICD with a single cysteine C317 that used lanthanide Tm^{3+} ions chelated to thiol-reactive N-[S-(2-pyridylthio)cysteaminyl]-EDTA[22,23]. Pairs of in-phase (blue) and anti-phase (red) ^{1}H-^{15}N IPAP TROSY peaks (after correcting the coupling constant in ^{1}H dimension) display the splitting in the ^{15}N dimension that contains $^{1}J_{NH}$ (94 Hz)[24] and RDC. Residues in the left spectrum show almost zero RDC, but residues G369 and G366 in the right spectrum show $|RDC| \geqq 2$. The spectra were collected at 35°C on a Bruker 900 MHz Avance spectrometer. RDC provides angular information relative to an external magnetic field, which is useful in determining the relative orientation of chemical bonds in a protein, regardless of spatial separation[25]. A total of 363 RDC values for the ^{1}H-^{15}N backbone amide bonds were obtained from three different single-cysteine α7nAChR TMD+ICD constructs (C219, C317, and C427).

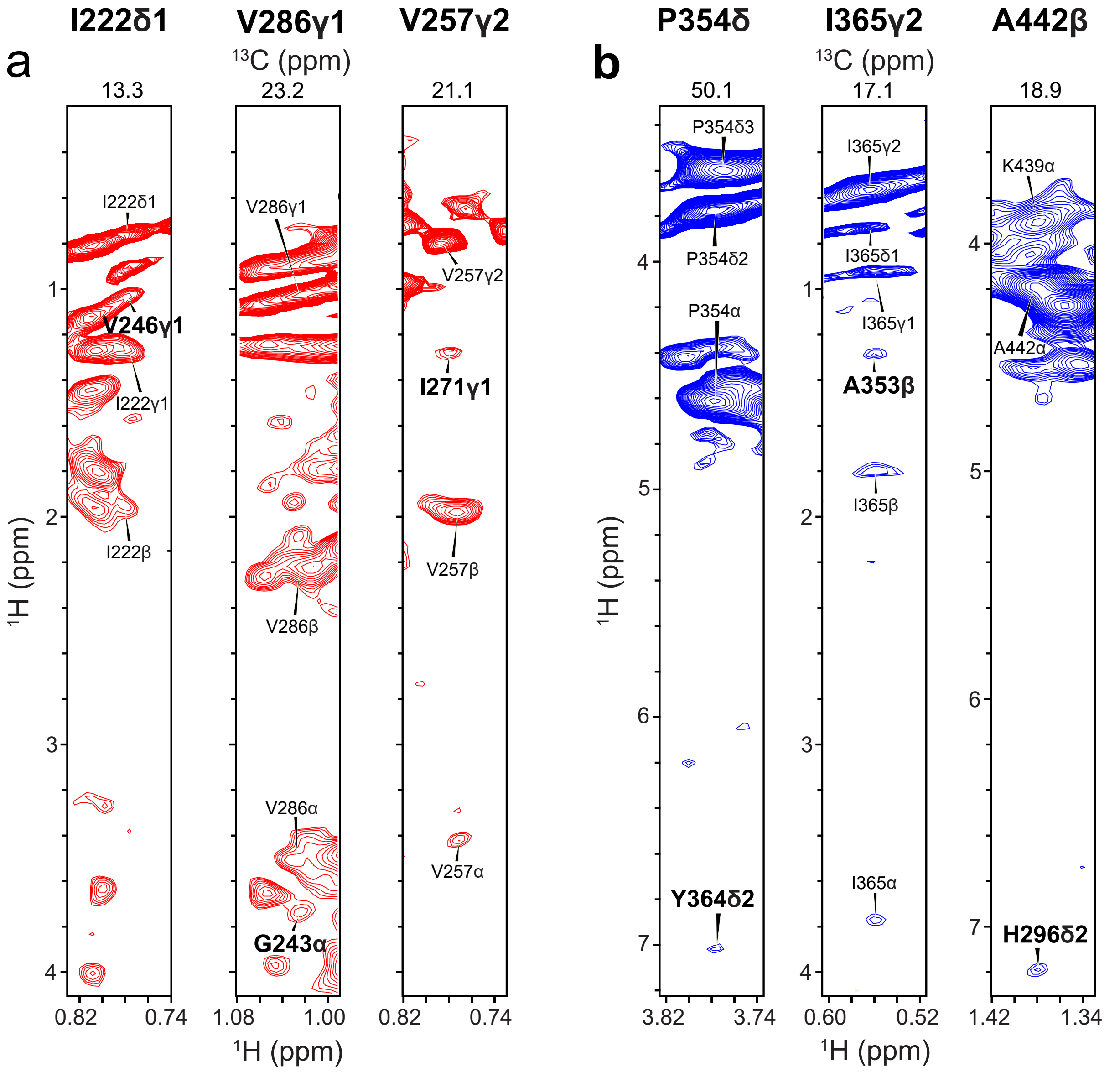

Supplementary Fig. 7. Representative intra-subunit NOEs for calculation and validation of α7nAChR TMD+ICD tertiary structures. Representative strip plots from a 3D ^{13}C-edited NOESY spectrum of the α7nAChR TMD+ICD (200 ms mixing time) that demonstrate long-range NOEs in (**a**) the TMD (red) and (**b**) the ICD (blue) between residues labeled in bold on the top and inside each strip plot. A total of 22 long-range TMD NOEs were used in the TMD+ICD structure calculations. Long-range ICD NOEs were used in structure validations.

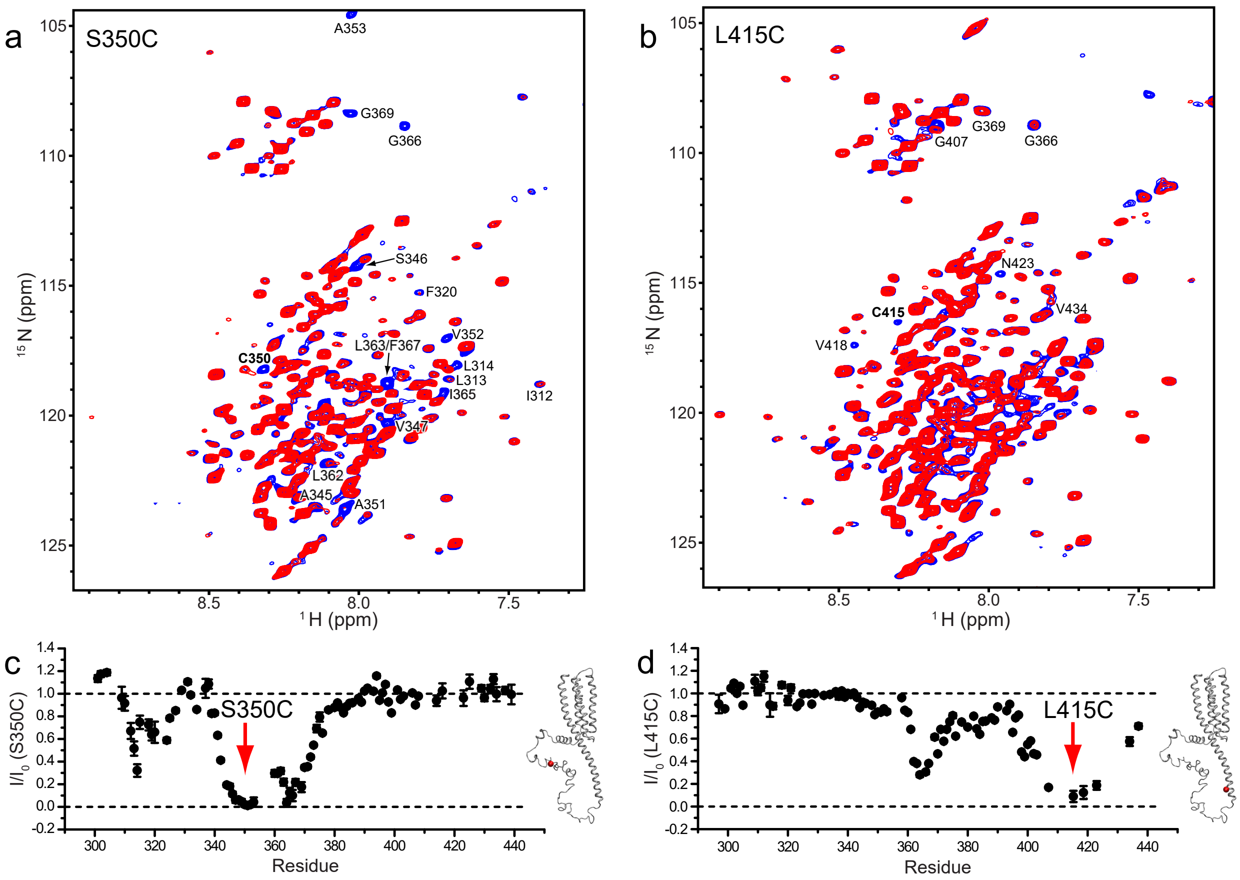

Supplementary Fig. 8. **Intra-subunit PREs for tertiary α7nAChR TMD+ICD structure determination**. Representative ^{1}H-^{15}N TROSY-HSQC PRE NMR spectra of single-cysteine α7nAChR TMD+ICD construct labeled with the paramagnetic MTSL at residues **(a)** S350C and **(b)** L415C in a paramagnetic state (red) and a diamagnetic state (blue) induced by the reducing agent ascorbic acid. Labeled residues show significant PREs, as reflected in their profound decrease of cross-peak intensities in the paramagnetic state. The intensity change provides the basis for quantifying distance restraints for structure determinations. The transverse relaxation rate (R_2) is dependent on the inverse sixth power of the distance ($1/R^6$) between the unpaired electron from an MTSL label and an observed proton[26,27,28,29]. (**c**) and (**d**) as well as (**e**)-(**m**) on the next page show quantified changes of normalized PRE (I/I_0) based on their corresponding NMR spectra as those shown in (a) and (b). A red arrow in each plot and a red sphere in each adjacent monomer structure highlight locations of individual paramagnetic MTSL-labeling residues, which include (**c**) S350C, (**d**) L415C, (**e**) V311C, (**f**) C317, (**g**) C335, (**h**) C442, (**i**) S358C, (**j**) C375, (**k**) C390, (l) C427, and (**m**) C435. I and I_0 represent NMR cross-peak intensities in the paramagnetic and diamagnetic states, respectively. Error bars represent the NMR measurement uncertainties derived from signal to noise of individual cross peaks (details in next page). Two horizontal dash lines at $I/I_0 = 0$ and $I/I_0 = 1$ mark the theoretical range of PRE effects (0 – maximum PRE and 1 – no PRE). Residues severely overlapped with other residues in the NMR spectra are excluded from the plots. Source data are provided as a Source data file. **(Continue in the next page)**

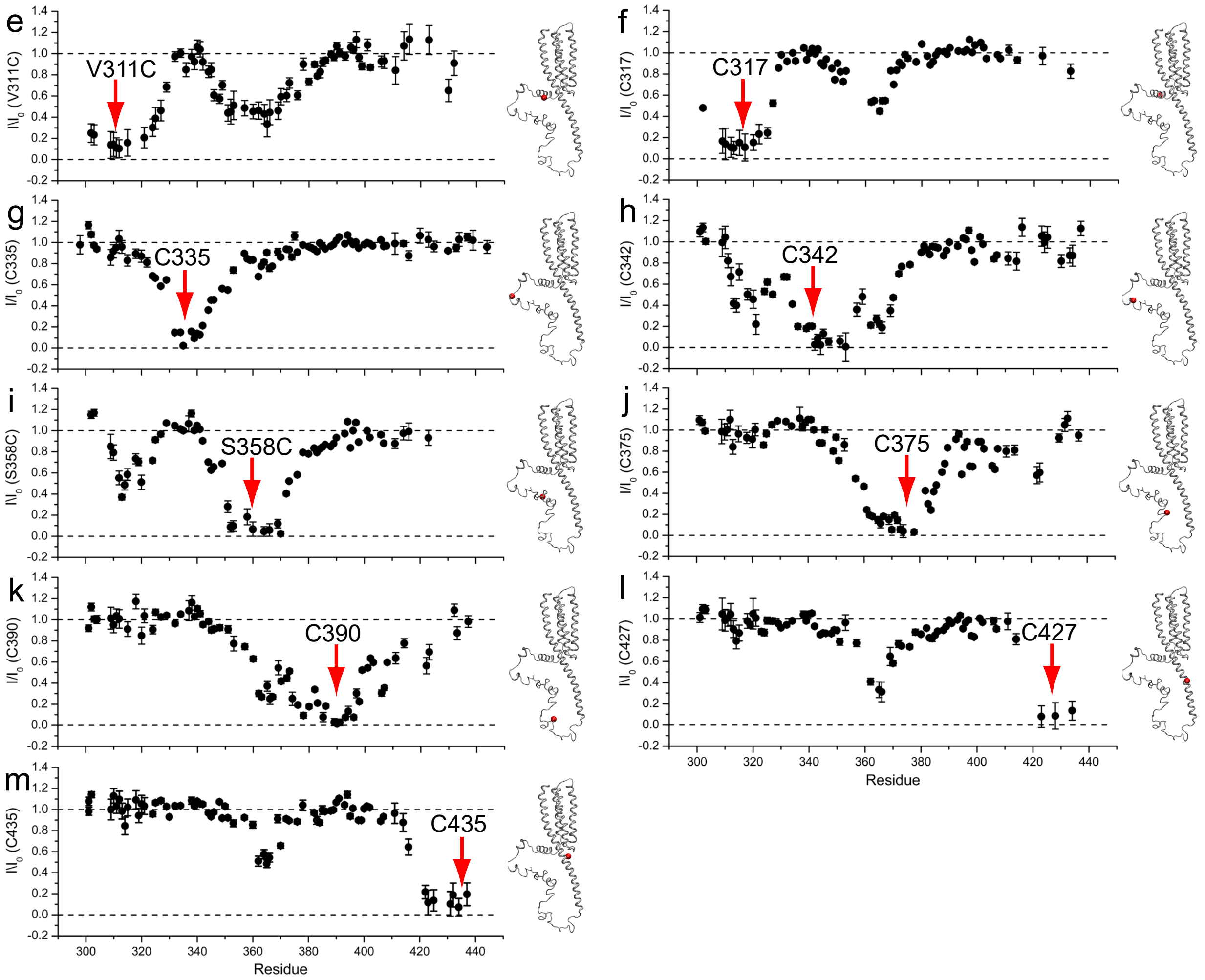

Supplementary Fig. 8. (Continuation from the previous page) (**e**)-(**m**) show the quantified changes of normalized PRE NMR intensity based on the corresponding NMR spectra as shown in (a) and (b). The location of the selected MTSL-labeling residue is highlighted by a red sphere in a monomer structure for clarity. Error bars in (c) –(m) represent uncertainties derived from signal to noise of individual cross peaks for each independent experiment using the equation:

$$\Delta\left(\frac{I}{I_0}\right) = \frac{I}{I_0}\sqrt{\left(\frac{\Delta I}{I}\right)^2 + \left(\frac{\Delta I_0}{I_0}\right)^2}$$

where I and I_0 are the cross-peak intensities in the respective paramagnetic and diamagnetic states, and ΔI and ΔI_0 are the corresponding noise levels in the NMR spectra. Data points in (**c**)-(**m**) are $I/I_0 \pm \Delta(I/I_0)$ of individual cross peaks in each pair of NMR spectra under paramagnetic and diamagnetic conditions based on an established data presentation method[18]. To demonstrate reproducibility, similar results were obtained from two independent samples for the construct C317. Source data are provided as a Source data file.

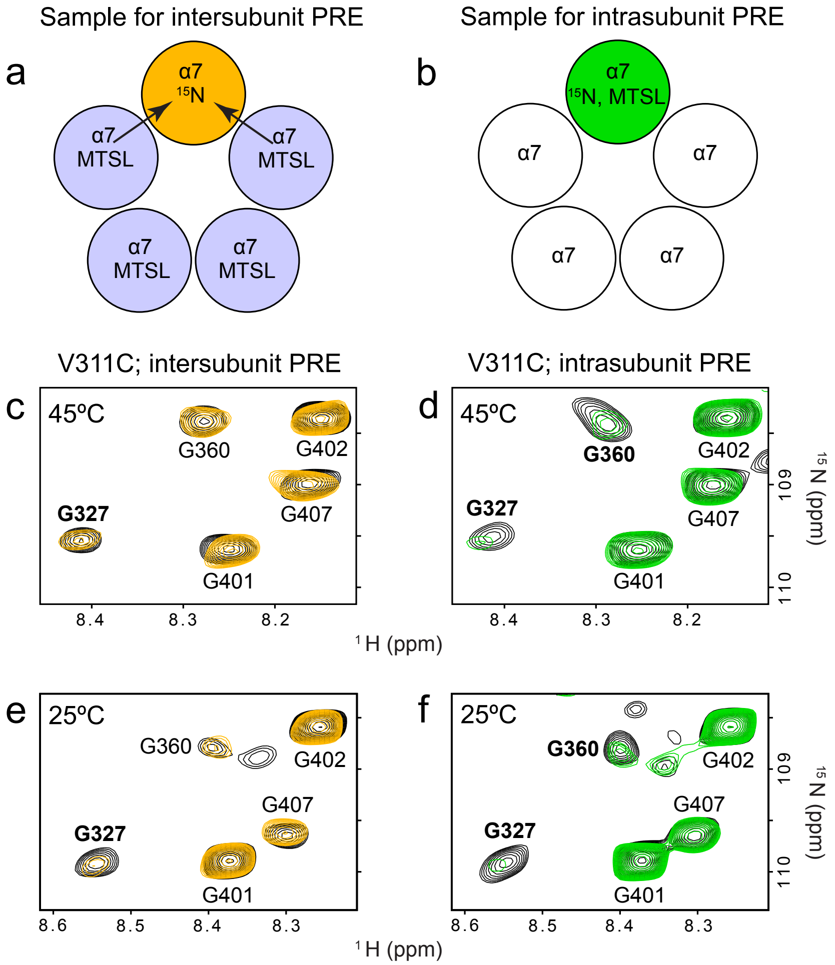

Supplementary Fig. 9. Identification of inter-subunit PREs for quaternary structure determination of α7nAChR TMD+ICD. Schematic presentation of NMR samples used to differentiate inter-subunit and intra-subunit PREs. Single cysteine TMD+ICD was expressed in two different media: #1) with uniform ^{15}N-labeling (NMR readout) and #2) no isotope labeling ("NMR invisible"). (**a**) To measure inter-subunit PREs, the TMD+ICD protein from #2 was labeled with MTSL (purple circle) and then mixed with #1 (orange circle) in a 4:1 molar ratio. Note that two MTSL-labeled subunits adjacent to the ^{15}N-labeled α7 subunit dominate the observed PREs. The remaining two MTSL-labeled subunits are too distal to contribute to the observed PREs. (**b**) To measure intra-subunit PREs, the uniformly ^{15}N-labeled TMD+ICD form #1 was labeled with MTSL (green circle) and mixed with the unlabeled protein from #2 (white circles) in a 1:4 molar ratio. (**c**) and (**d**) Representative ^{1}H-^{15}N TROSY-HSQC PRE NMR spectra of a glycine region, collected at 45 ºC, from samples in (a) and (b), respectively. Specifically, the spectra resulted from single-cysteine (V311C) α7nAChR TMD+ICD samples (70 µM of ^{15}N α7) in the presence (black) and absence (orange or green) of the reducing agent ascorbic acid (2.8 mM). Residues showing significant inter-subunit and intra-subunit PREs are marked in bold. (**e**) and (**f**) ^{1}H-^{15}N TROSY-HSQC PRE NMR spectra of the same samples used for (c) and (d), respectively, but collected at 25 ºC. All of the spectra were collected on an 18.8 tesla Bruker Avance spectrometer. Additional representative spectra showing inter-subunit PREs for quaternary structure determination of α7nAChR TMD+ICD are provided in next page.

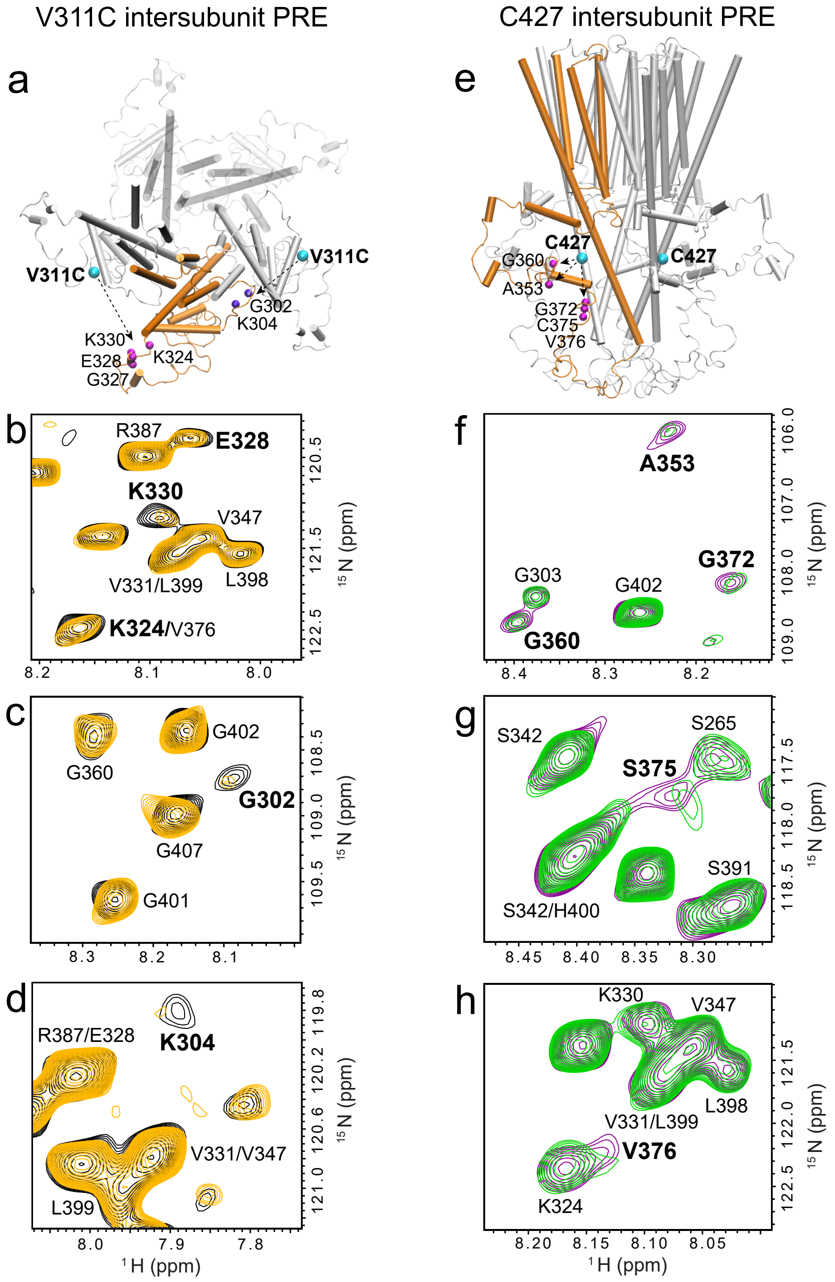

Supplementary Fig. 9. (A continuation from the previous page) **Additional representative spectra showing inter-subunit PREs for quaternary structure determination of α7nAChR TMD+ICD**. (**a**) Top view showing two residue-groups (magenta, violet) experiencing inter-subunit PRE resulting from the MTSL-labeled V311C (cyan) in two adjacent subunits. (**b**)-(**d**) Representative regions of ^{1}H-^{15}N TROSY-HSQC PRE NMR spectra collected at 45ºC. Residues labeled in bold demonstrate PRE due to the MTSL-labeled V311C. (**e**) Side view showing residues (magenta) experiencing inter-subunit PRE resulting from the MTSL-labeled C427 in an adjacent subunit. (**f**)-(**h**) Representative regions of ^{1}H-^{15}N TROSY-HSQC PRE NMR spectra collected at 45ºC. Residues labeled in bold demonstrate PRE due to the MTSL-labeled C427.

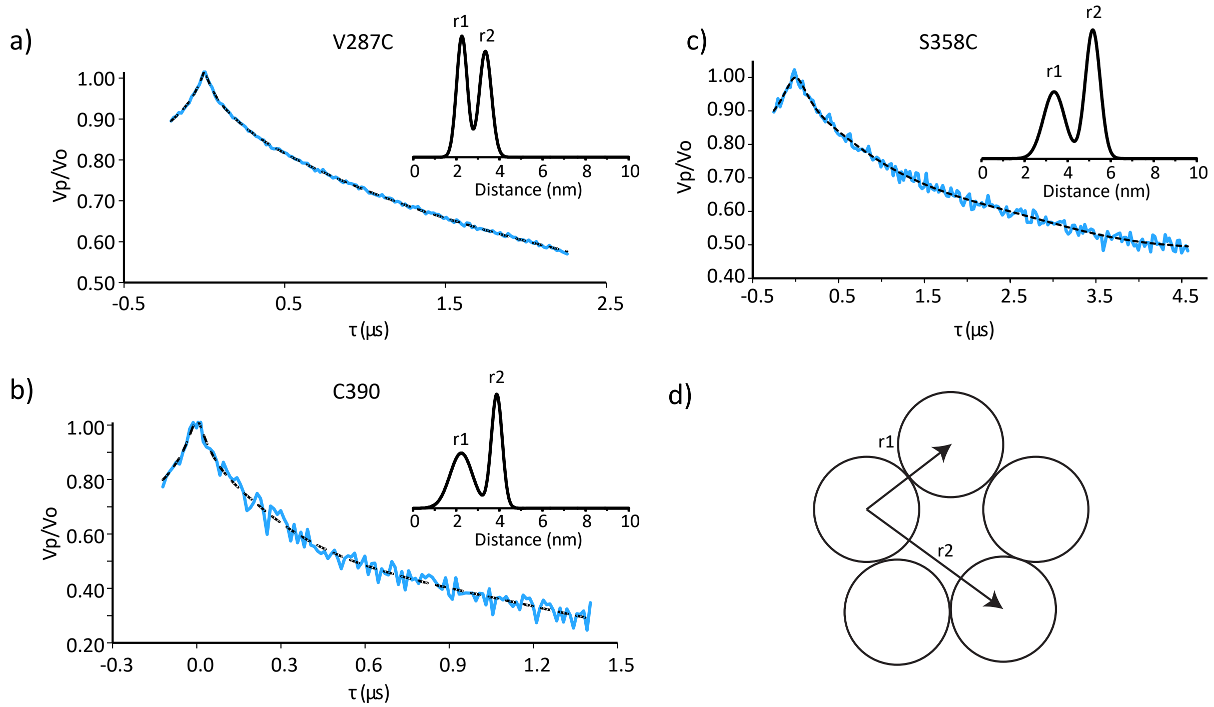

Supplementary Fig. 10. **Representative DEER ESR data for quaternary structure restraints.** Time domain signals (blue lines) and best fits (black dashed lines) of full-length α7nAChR labeled with MTSL at residues (**a**) V287C, (**b**) C390, and (**c**) S358C**.** Insets show the resulting adjacent (r1) and across (r2) distance distributions obtained from the DD[30]. DD was chosen as it can fit the DEER ESR data without the need for background subtraction. This eliminates artifacts and biases due to manual background subtraction. The number of Gaussians used in the DD model resulted from the Akaike information criterion corrected for finite sample size (AICc). The AICc parameter depends on two components of a fit, the RMSD of a fit and the number of parameters used to fit the data. When AICc is minimized, this represents a fit in which provides a low RMSD without having too many input parameters to fit the data. From this, a two Gaussian model was found to be most optimal. The average distance and error of the two Gaussians serve as input parameters for Rosetta calculations. (**d**) r1 and r2 distances marked in the top view of a cartoon α7nAChR pentamer. (**a**) Q-band DEER of α7nAChR V287C in SMA-asolectin nanodiscs resulted in two major distance distributions centered at r1=2.3 ± 0.3 nm and r2=3.3 ± 0.2 nm. The data were collected at 80 K with DEER parameters: $(\pi)_{v1}$ = 24 ns, $(\pi)_{v2}$ = 24 ns, dt = 16 ns, n = 155. (**b**) X-band DEER of α7nAChR C390 in asolectin liposomes results in r1= 2.2 ± 0.2 nm and r2=3.9 ± 0.2 nm. DEER parameters were $(\pi)_{v1}$ = 32 ns, $(\pi)_{v2}$, = 16 ns, dt = 12 ns, n = 128, and temperature of 80 K. (**c**) Q-band DEER of S358C in asolectin liposomes results in r1= 3.3 ± 0.3 nm and r2= 5.2 ± 0.2 nm. DEER parameters were $(\pi)_{v1}$ = 16 ns, $(\pi)_{v2}$, = 24 ns, dt = 24 ns, n = 202, and temperature = 50 K. Distances are reported as mean ± 95% confidence interval. Source data are provided in a Source data file.

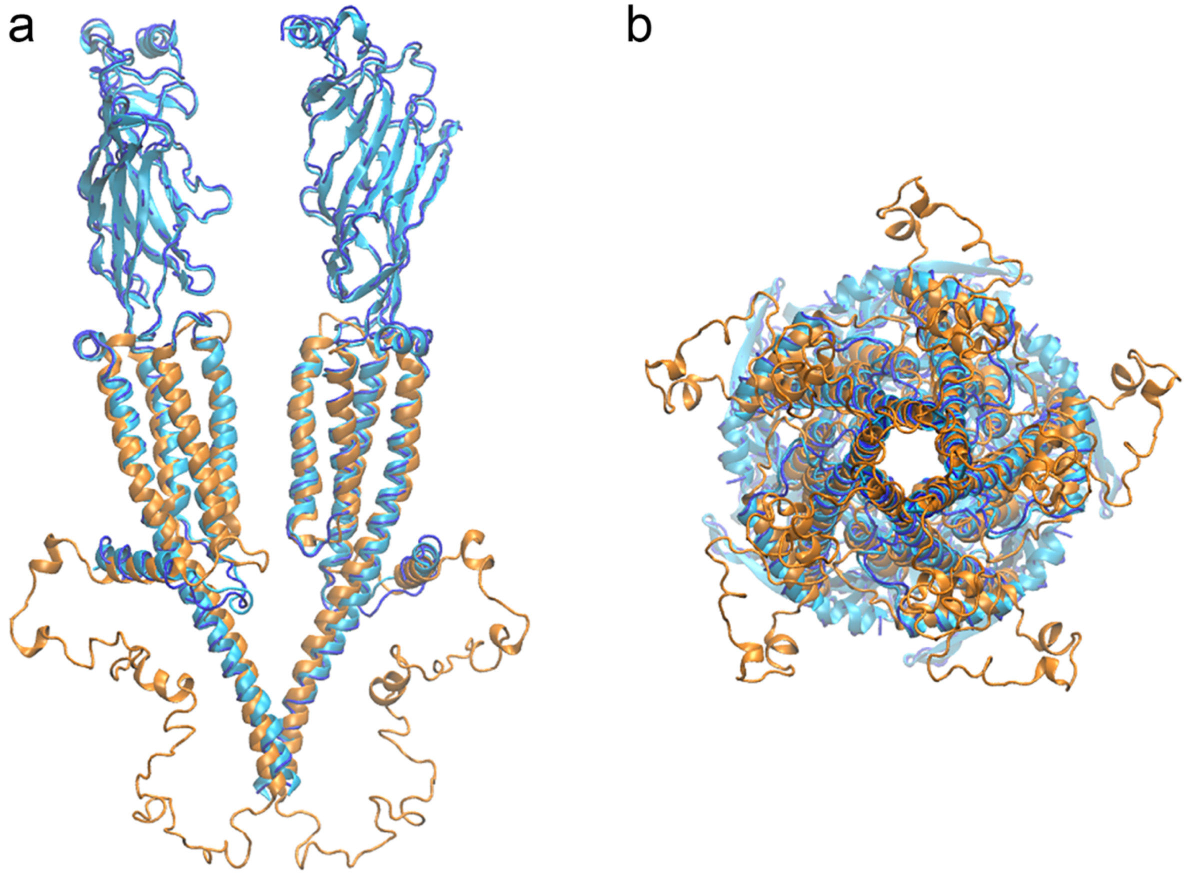

Supplementary Fig. 11. Comparison of the new α7nAChR TMD+ICD structure (orange, PDBID: 7RPM) **with the Cryo-EM structures of apo α7nAChR** (blue, PDBID: 7EKI)[31] and **α-bungarotoxin-bound resting-state α7nAChR** (cyan, PDBID: 7KOO)[32]. (**a**) A side view of the aligned TMD and ICD structures. For clarity, only two subunits are shown. (**b**) A bottom view of the aligned MA and MX helices from the three structures. The structure alignment used the regions of TM1-TM3 (L209-Y295), MX helix (W308-R321), and MA-TM4 helix (D410-P469). Pairwise backbone RMSD values (TMD+ICD vs. Cryo-EM structure) were calculated for all regions ($RMSD_{7EKI}$ = 2.66 Å; $RMSD_{7KOO}$ = 2.47 Å) and for the helical regions ($RMSD_{7EKI}$ = 2.21 Å; $RMSD_{7KOO}$= 1.94 Å), which included L209-L231 (TM1), G237-I260 (TM2), P269-Y295 (TM3), W308-R321 (MX), and D410-P469 (MA-TM4). Structure alignment and RMSD calculations were performed using VMD[33].

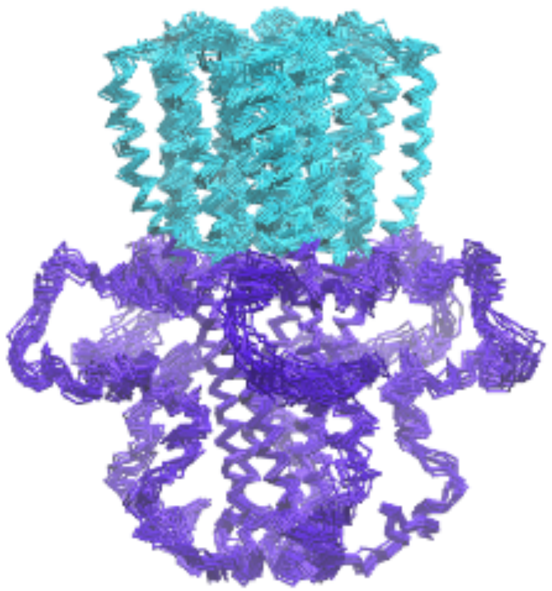

Supplementary Fig. 12. A stereo image of the TMD (cyan) + ICD (violet) structure. The superimposed backbone traces are from the 15 lowest energy structures. Their atomic coordinates and structural restraints have been deposited to the Protein Data Bank with accession code: 7RPM.

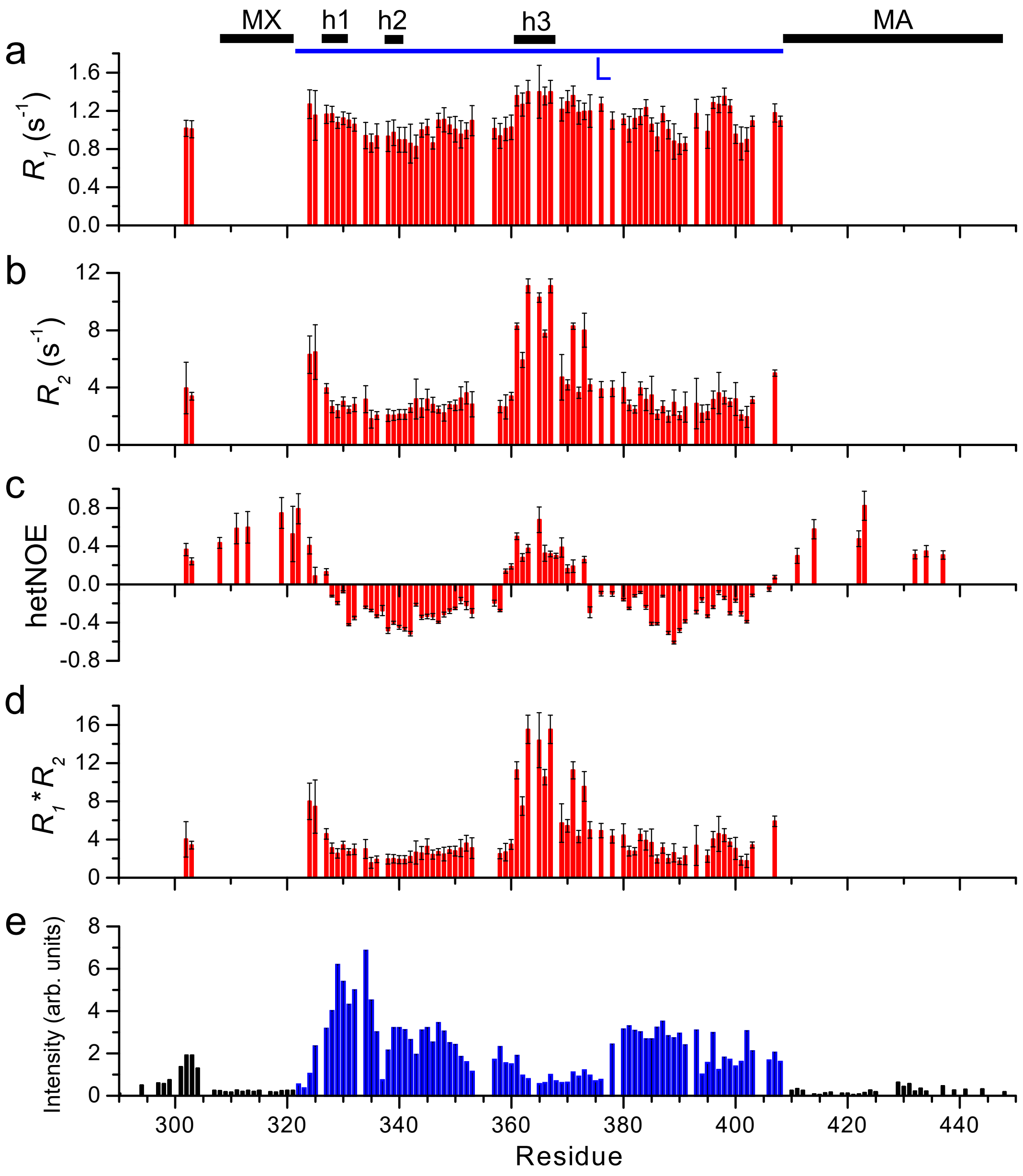

Supplementary Fig. 13. NMR measurement of α7nAChR TMD+ICD dynamics. The backbone dynamics of TMD+ICD were determined through solution NMR residue specific (**a**) ^{15}N spin-lattice relaxation rate R_1, (**b**) ^{15}N spin-spin relaxation rate R_2, and (**c**) ^{15}N-(^{1}H) heteronuclear NOE. R_1 and R_2 were obtained from fitting of the measured cross-peak intensities over relaxation delay, t, to the exponential decay functions $I(t) \propto e^{-R1t}$ and $I(t) \propto e^{-R2t}$; error bars in (a) and (b) indicate the corresponding fitting errors. The hetNOE values in (c) were obtained from the peak intensity ratios in the presence and absence of proton saturation. Error bars (ΔNOE) reflect uncertainties resulting from NMR signal to noise:

$$\Delta NOE = NOE\sqrt{\left(\frac{\Delta I_{sat}}{I_{sat}}\right)^2 + \left(\frac{\Delta I_{unsat}}{I_{unsat}}\right)^2},$$

where I_{sat} and I_{unsat} are the peak intensity with and without proton saturation, respectively, and ΔI_{sat} and ΔI_{unsat} represent the corresponding noise levels in the NMR spectra[34]. Data in (a) – (c) were collected at 45 °C on an NMR spectrometer operating at 16.4 tesla. A notable feature from (a)-(c) is that most residues in loop L (marked on the top of the figure) exhibit negative NOE (c) and low R_2 values (b), a fingerprint of flexible disordered regions[35]. Residues near the h3 helix, however, show positive NOE (c) and relatively high R_2 values (b), an indication of a more ordered structural region. R_1R_2 products are sensitive to motion in μs-ms timescale[36]. (**d**) Higher R_1R_2 near helix h3 suggests a possible μs-ms motion in the h3 region. Consistent with findings in (a)-(d), (**e**) intensity (peak height) distribution of TMD+ICD residues obtained from a HNCOCA NMR spectrum also shows profound backbone dynamics difference between residues near the h3 helix and the rest of the residues in loop L. Missing bars in (a)-(e) are due to prolines, overlapping residues, or residues in MX and MA helical regions where NMR signals are too weak to be used for reliable determinations of R_1, R_2, and ^{15}N-(^{1}H) hetNOE in (a)-(c). Source data are provided as a Source Data file.

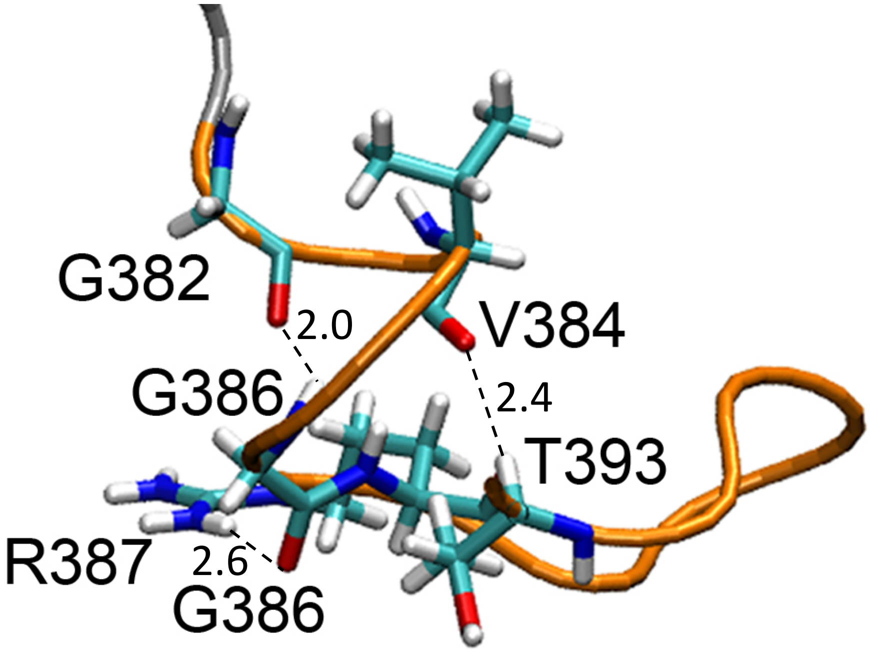

Supplementary Fig. 14. Interactions stabilizing the GXXXG motif. In the segment of [382GVVCG386], G382 and G386 are in close contact through their respective carbonyl oxygen and amide (2.0 Å), forming a weak hydrogen bond. Other interactions are observed in G386-R387 (2.6 Å) and V384-T393 (2.4 Å). These interactions contribute to stabilization of the GXXXG motif.

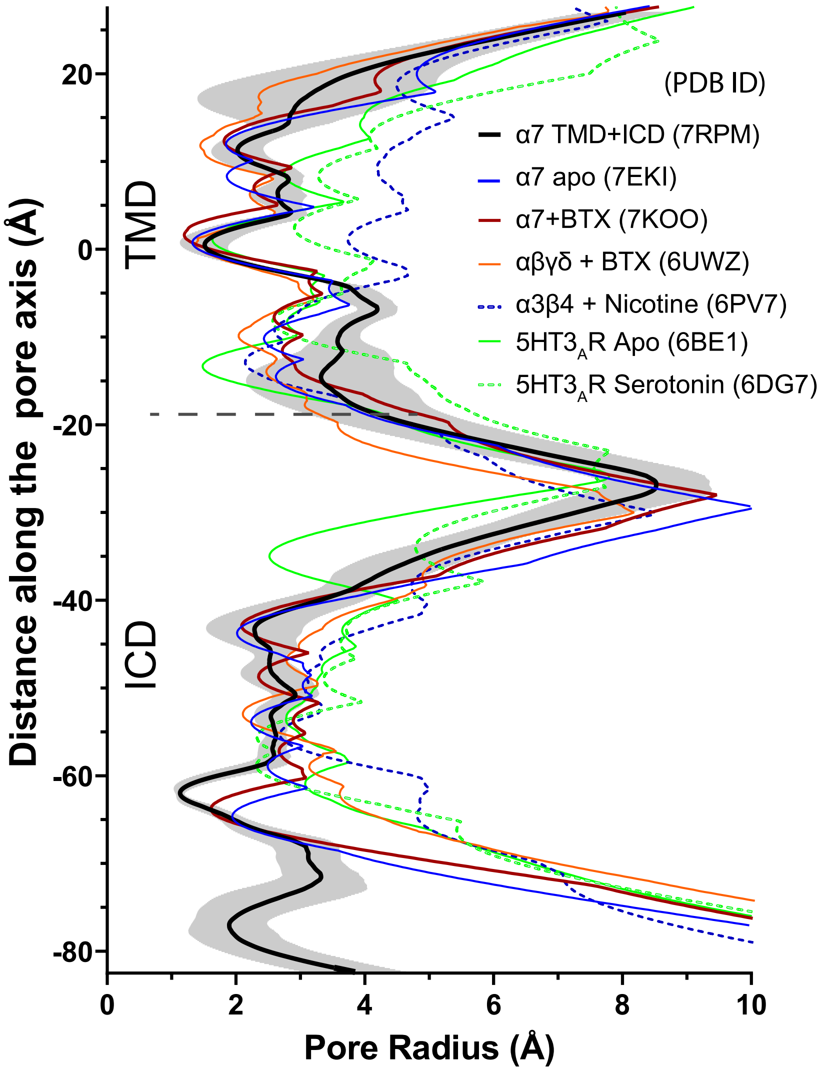

Supplementary Fig. 15. Pore profile comparisons of the α7nAChR TMD+ICD with other Cys-loop receptors. The averaged pore profile from 15 structures of the α7nAChR TMD+ICD (black; the gray shadow shows the standard deviation) is compared with the pore profiles of apo α7nAChR (blue, PDBID: 7EKI)[31], α-bungarotoxin-bound α7nAChR (crimson, PDBID: 7KOO)[32], α-bungarotoxin-bound αβγδAChR (orange, PDBID: 6UWZ)[37], nicotine-bound α3β4nAChR (blue dash, PDBID: 6PV7)[38], apo-5HT$_3$$_A$R (green, PDBID: 6BE1)[6], and serotonin-bound 5HT$_{3A}$R (green dash, PDBID: 6DG7)[39]. The pore radius is calculated by HOLE[40] and plotted as a function of distance along the pore axis. The transmembrane domain (TMD) and intracellular domain (ICD) are separated by a dashed line. The pore profile of the newly determined α7nAChR TMD+ICD structure matches reasonably well with the pore profiles of the apo α7nAChR (blue) and α-bungarotoxin-bound α7nAChR (crimson). The extra length of the pore profile shown around -80 from the new TMD+ICD structure is contributed by residues in loop L (Fig 2) that are missing in previously published structures. Source data are provided as a Source Data file.

Supplementary Table 1. NMR and ESR experimental restraints for α7nAChR structure calculations (number of restraints per subunit).

Experimental restraints	TMD (116 residues)	ICD (148 residues)	TMD+ICD (264 residues)
NMR chemical shifts [a]			
N	93 (0.80)[b]	126 (0.85)	219 (0.83)
Cα	102 (0.88)	147 (0.99)	249 (0.94)
C	77 (0.66)	110 (0.74)	187 (0.71)
Cβ	80 (0.69)	65 (0.44)	145 (0.55)
Hα	97 (0.84)	84 (0.57)	181 (0.69)
NMR NOE distance restraints			824
Intra-residue			476
Inter-residue			348
Sequential ($\|i-j\| = 1$)			254
Medium-range ($2 \le \|i-j\| \le 4$)			72
Long-range ($\|i-j\| \ge 5$)			22
Inter-subunit			0
Hydrogen bonds [c]		110	266
NMR RDC restraints [d]		271	363
NMR PRE distance restraints [e]		615	615
Class 1		42	42
Class 2		294	294
Class 3		266	266
Additional inter-subunit [f]		13	13
DEER ESR distance restraints		10	18

[a] Chemical shifts were used for generating 3- and 9-residues fragments using CS-Rosetta[20] and for secondary structure determinations using TALOS+[21].

[b] Fractions of assigned residues = the number of assigned residues/a total number of residues

[c] Hydrogen bond (helical) distance restraints were used for residues 291-296, 307-321, 361-369, and 409-449 in the ICD structure calculations and for residues 209-231, 238-260, 270-296, 307-321, 361-369, and 409-469 in the TMD+ICD structure calculations.

[d] RDC restraints were only used for generating 3- and 9-residues fragments using CS-Rosetta[20].

[e] Class 1, 2, and 3 of PRE distance restraints are defined as follows:

Class 1: $r \le 16$ Å ($I/I_0 \le 0.15$)

Class 2: $16 < r < 26$ Å ($0.15 < I/I_0 < 0.85$)

Class 3: $r \ge 26$ Å ($I/I_0 \ge 0.85$)

I and I_0 are NMR peak intensities in paramagnetic and diamagnetic states, respectively.

[f] Including two ^{19}F NMR distance restraints from our study published previously[41].

Supplementary Table 2. Statistics for the 15 refined α7nAChR TMD+ICD structures.

**Structure statistics** [a]	
Q-factors	
Q^{DEER}	0.11 ± 0.03
Q^{NOE}	0.09 ± 0.10
Q^{PRE}	0.21 ± 0.08
$Q^{PRE}{}_{free}$ [b]	0.12 ± 0.03
Violations	
NMR NOE distance restraints (Å)	0.13 ± 0.14
NMR PRE distance restraints (Å)	1.32 ± 0.52
DEER ESR distance restraints (Å)	1.85 ± 0.49
Deviations from idealized geometry	
Bond lengths (Å)	0.0035 ± 0.0001
Bond angles (º)	0.88 ± 0.01
Impropers (º)	0.36 ± 0.01
Rotamer Outliers (%)	0.12 ± 0.20
Clash score	1.8 ± 0.6
MolProbity score	0.98 ± 0.15
Ramachandran plot	
favored (%)	98.1 ± 0.8
outliers (%)	0.00 ± 0.00
Average pairwise r.m.s. deviation (Å)	
Heavy:	
(Residues 209-472; all residues)	2.15 ± 0.55
(Residues 209-231, 238-260, 270-296, 308-321, 409-469; helical regions)	1.49 ± 0.36
Backbone	
(Residues 209-472; all residues)	2.58 ± 0.52
(Residues 209-231, 238-260, 270-296, 308-321, 409-469; helical regions)	2.00 ± 0.34

[a] The reported are Mean ± SD. Structure statistics were from 15 structures deposited in PDB. Phenix[15] and MolProbity[42] were used for structure statistics. Xplor-NIH[43] was used for evaluating "impropers" because it was not reported by Phenix and MolProbity.

[b] 56 PRE restraints (~10% of total PRE restraints) that were excluded in structure calculations were used for the $Q^{PRE}{}_{free}$ calculation.

Supplementary Table 3. Comparisons of intracellular domain (ICD) sequences of selected Cys-loop receptors. The percentage of sequence identity is calculated using Clustal Omega[44]

Name	Uniprot ID	Total # of ICD residues	ICD Sequence	% sequence identity (relative to ACHA7)
ACHA7_HUMAN	P36544	148	296HHHDPDGGKMPK**WTRVILLNWCAWFL**RMKRPGEDKVRPACQHKQRRCSLASVEMSAVAPPPASNGNLLYIGFRGLDGVHCVPTPDSGVVCGRMACSPTHDEHLLHGGQPPEGD**PDLAKILEEVRYIANRFRCQDESEAVCSEWKFAAC**	
ACHA4_HUMAN	P43681	266	303HHRSPRTHTMPT**WVRRVFLDIVPRLL**LMKRPSVVKDNCRRLIESMHKMASAPRFWPEPEGEPPATSGTQSLHPPSPSFCVPLDVPAEPGPSCKSPSDQLPPQQPLEAEKASPHPSPGPCRPPHGTQAPGLAKARSLSVQHMSSPGEAVEGGVRCRSRSIQYCVPRDDAAPEADGQAAGALASRNTHSAELPPPDQPSPCKCTCKKEPSSVSPSATVKTRSTKAPPPHLPLS**PALTRAVEGVQYIADHLKAEDTDFSVKEDWKYVAM**	**MX 28.6**<br>**MA 34.3**<br>All 29.7
ACHB2_HUMAN	P17787	135	297HHRSPTTHTMAP**WVKVVFLEKLPALL**FMQQPRHHCARQRLRLRRRQREREGAGALFFREAPGADSCTCFVNRASVQGLAGAFGAEPAPVAGPGRSGEPCG**CGLREAVDGVRFIADHMRSEDDDQSVSEDWKYVAM**	**MX 28.6**<br>**MA 31.4**<br>All 24.5
ACHA3_HUMAN	P32297	145	298HYRTPTTHTMPS**WVKTVFLNLLPRVM**FMTRPTSNEGNAQKPRPLYGAELSNLNCFSRAESKGCKEGYPCQDGMCGYCHHRRIKISNFSANLTRSSSSESVDAVLSLSALSPE**IKEAIQSVKYIAENMKAQNEAKEIQDDWKYVAM**	**MX 21.4**<br>**MA 27.3**<br>All 23.0
ACHB4_HUMAN	P30926	137	299HHRSPSTHTMAP**WVKRCFLHKLPTFL**FMKRPGPDSSPARAFPPSKSCVTKPEATATSTSPSNFYGNSMYFVNPASAASKSPAGSTPVAIPRDFWLRSSGRF**RQDVQEALEGVSFIAQHMKNDDEDQSVVEDWKYVAM**	**MX 21.4**<br>**MA 34.3**<br>All 28.2
ACHA_TETCF	P02710	106	298HHRSPSTHTMPQ**WVRKIFIDTIPNVM**FFSTMKRASKEKQENKIFADDIDISDISGKQVTGEVIFQTPLIKN**PDVKSAIEGVKYIAEHMKSDEESSNAAEEWKYVAM**	**MX 21.4**<br>**MA 37.1**<br>All 25.3
5HT3A_MOUSE	P23979	124	310VHKQDLQRPVPD**WLRHLVLDRIAWIL**CLGEQPMAHRPPATFQANKTDDCSGSDLLPAMGNHCSHVGGPQDLEKTPRGRGSPLPPPREAS**LAVRGLLQELSSIRHFLEKRDEMREVARDWLRVGY**	**MX 42.9**<br>**MA 23.3**<br>All 22.6
GBRA1[#]_HUMAN	P14867	81	310FTKRGYAWDGKSVVPEKPKKVKDPLIKKNNTYAPTATSYTPNLARGDPGLATIAKSATIEPKEVKPETK**PPEPKKTFNSVS**	**MA 27.3**<br>All 11.9
GLRA1[#]_HUMAN	P23415	87	307VSRQHKELLRFRRKRRHHKSPMLNLFQEDEAGEGRFNFSAYGMGPACLQAKDGISVKGANNSNTTNP**PPAPSKSPEEMRKLFIQRAK**	**MA 26.3**<br>All 17.1

* MX and MA helices in the ICD are underlined and colored in red and purple, respectively.
[#] The anion channels, $GABA_A$Rs and GlyRs, do not have MX helix.

Supplementary Table 4. The primers used for the TMD+ICD constructs containing a single unpaired cysteine.

Oligo name	5′ to 3′ sequences
**TMD vector_fwd**	gaccgcctgtgcctcatg
**TMD vector_rev**	gtcggggtcgtggtggtg
**ICD_fwd**	accaccaccacgaccccgacgggggcaagatgcccaag
**ICD_rev**	gccatgaggcacaggcggtccaccacacaggcggcgaa
**C219A**	aacctgctgatccccgctgtgctcatctccgc
**C317S**	tccttctgaactggagcgcgtggttcctg
**C335S**	tgcgcccggccagccagcacaag
**C342S**	agcagcggcgcagcagcctggcc
**C375S**	gacggcgtgcacagtgtcccgaccc
**C385S**	ctctggggtagtgagtggccgcatggc
**C390S**	gccgcatggccagctcccccacg
**C427S**	caaccgcttccgcagccaggacgaaag
**C435S**	gcgaggcggtcagcagcgagtgg
**C443S**	gaagttcgccgccagtgtggtggaccg
**C449A**	ggtggaccgcctggccctcatggccttc
**C460A**	ggtcttcaccatcatcgccaccatcggcatcctg
**A272C**	aacatccgattcggtaccattgatatgccagtacttcgcc
**V287C**	cgtgggcctctcggtgtgcgtgacagtgatcgtgc
**V311C**	atgcccaagtggaccagatgcatccttctgaactggtg
**S350C**	agtgtggagatgtgcgccgtggcgc
**S358C**	cgccgcccgcctgcaacgggaac
**L415C**	cggacttggccaagatctgcgaggaggtccgctacat
**E437C**	gaggcggtcagcagctgctggaagttcgccgcc

Supplementary Table 5. NMR samples, experiments, and most relevant acquisition parameters

Backbone ^{1}H, ^{15}N, ^{13}C chemical shift assignment					
**Samples 1-10**: 0.24 mM ^{15}N-, ^{13}C-labeled WT α7nAChR TMD-ICD, 5 mM sodium acetate pH4.7, 66 mM (1.5%) LDAO, 25 mM NaCl; varied temperatures: 298, 308, 318 K					
**NMR experiment**	**Spectral width (ppm)**	**Data points in time domain**	**Number of scans**	**Data collection time**	**NMR field strength and other notes**
2D ^{1}H-^{15}N TROSY-HSQC	13×23 ppm ($^{1}H_{N}$×^{15}N)	1024×200 ($^{1}H_{N}$×^{15}N)	48	3 h	18.8 T, 298 K
3D BEST-HNCA	13×23×23 ppm ($^{1}H_{N}$×^{15}N×^{13}C)	1024×48×88 ($^{1}H_{N}$×^{15}N×^{13}C)	48	1 d 8 h	18.8 T, 298 K
3D BEST-HN(CO)CA	13×23×23 ppm ($^{1}H_{N}$×^{15}N×^{13}C)	1024×48×88 ($^{1}H_{N}$×^{15}N×^{13}C)	64	1 d 20 h	18.8 T, 298 K
3D TROSY-CBCACONH	13×23×57 ppm ($^{1}H_{N}$×^{15}N×^{13}C)	1024×40×112 ($^{1}H_{N}$×^{15}N×^{13}C)	16	22 h	18.8 T, 298 K
3D BEST-HNCO	13×23×12 ppm ($^{1}H_{N}$×^{15}N×^{13}C)	1024×48×64 ($^{1}H_{N}$×^{15}N×^{13}C)	32	16 h	18.8 T, 298 K
2D ^{1}H-^{15}N TROSY-HSQC	13×23 ppm ($^{1}H_{N}$×^{15}N)	1024×200 ($^{1}H_{N}$×^{15}N)	24	1.5 h	18.8 T, 318 K
3D BEST-HNCA	13×23×23 ppm ($^{1}H_{N}$×^{15}N×^{13}C)	1024×44×80 ($^{1}H_{N}$×^{15}N×^{13}C)	32	18 h	18.8 T, 318 K
3D BEST-HN(CO)CA	13×23×23 ppm ($^{1}H_{N}$×^{15}N×^{13}C)	1024×44×80 ($^{1}H_{N}$×^{15}N×^{13}C)	32	18 h	18.8 T, 318 K
3D TROSY-CBCACONH	13×23×57 ppm ($^{1}H_{N}$×^{15}N×^{13}C)	1024×40×112 ($^{1}H_{N}$×^{15}N×^{13}C)	16	22 h	18.8 T, 318 K
3D BEST-HNCO	13×23×12 ppm ($^{1}H_{N}$×^{15}N×^{13}C)	1024×44×56 ($^{1}H_{N}$×^{15}N×^{13}C)	16	6 h	18.8 T, 318 K
2D ^{1}H-^{15}N TROSY-HSQC	13×23 ppm ($^{1}H_{N}$×^{15}N)	1024×200 ($^{1}H_{N}$×^{15}N)	24	1.5 h	18.8 T, 308 K
**Samples 11 - 14**: 0.25 mM ^{15}N-, ^{13}C-labeled α7nAChR C317 TMD+ICD, 5 mM sodium acetate pH 4.7, 44 mM (1%) LDAO, 25 mM NaCl; temperature: 318 K					
**NMR experiment**	**Spectral width (ppm)**	**Data points in time domain**	**Number of scans**	**Data collection time**	**NMR field strength/ other notes**
2D ^{1}H-^{15}N TROSY-HSQC	13×23 ppm ($^{1}H_{N}$×^{15}N)	1024×176 ($^{1}H_{N}$×^{15}N)	48	3 h	18.8 T
3D TROSY-HNCA	12×23×28 ppm ($^{1}H_{N}$×^{15}N×^{13}C)	1024×36×72 ($^{1}H_{N}$×^{15}N×^{13}C)	80	2 d 18 h	18.8 T
3D TROSY-HN(CO)CA	12×23×28 ppm ($^{1}H_{N}$×^{15}N×^{13}C)	1024×36×72 ($^{1}H_{N}$×^{15}N×^{13}C)	96	3 d 18 h	18.8 T
3D TROSY-HNCACB	12×23×56 ppm ($^{1}H_{N}$×^{15}N×^{13}C)	1024×36×104 ($^{1}H_{N}$×^{15}N×^{13}C)	56	2 d 21 h	16.4 T
3D TROSY-CBCACONH	12×23×56 ppm ($^{1}H_{N}$×^{15}N×^{13}C)	1024×36×104 ($^{1}H_{N}$×^{15}N×^{13}C)	56	2 d 22 h	16.4 T
3D TROSY-HNCO	12×23×12 ppm ($^{1}H_{N}$×^{15}N×^{13}C)	1024×56×64 ($^{1}H_{N}$×^{15}N×^{13}C)	32	1 d 13 h	16.4 T
**Sample 15-16**: 0.35 mM ^{15}N-, ^{13}C-labeled α7nAChR C317 TMD-ICD, 5 mM sodium acetate pH4.7, 44 mM (1%) LDAO, 25 mM NaCl; temperature: 318 K					

2D ^{1}H-^{15}N TROSY-HSQC	13×23 ppm ($^{1}H_N$×^{15}N)	1024×176 ($^{1}H_N$×^{15}N)	48	3 h	18.8 T
3D TROSY-HNCA	12×21×26 ppm ($^{1}H_N$×^{15}N×^{13}C)	1024×36×96 ($^{1}H_N$×^{15}N×^{13}C)	80	3 d 16 h	18.8 T
3D TROSY-HN(CO)CA	12×21×26 ppm ($^{1}H_N$×^{15}N×^{13}C)	1024×36×96 ($^{1}H_N$×^{15}N×^{13}C)	64	2 d	18.8 T
**Sample 17**: 0.25 mM ^{15}N-, ^{13}C-labeled α7nAChR C317 TMD-ICD, 5 mM sodium acetate pH4.7, 44 mM (1%) LDAO, 25 mM NaCl; temperature: 303 K					
3D BEST-HNCA	12×23×27 ppm ($^{1}H_N$×^{15}N×^{13}C)	1024×48×104 ($^{1}H_N$×^{15}N×^{13}C)	16	13 h	21.1 T
Determination of temperature slope of $^{1}H_N$ chemical shift (for secondary structure determination)					
**Sample 18**: 0.30 mM ^{15}N-, ^{13}C-labeled α7nAChR C317 TMD-ICD, 5 mM sodium acetate pH4.7, 66 mM (1.5%) LDAO, 25 mM NaCl; temperatures: 283, 288, 293, 298, 303, 308, 313, and 318 K					
2D ^{1}H-^{15}N TROSY-HSQC	13×23 ppm ($^{1}H_N$×^{15}N)	2048×200 ($^{1}H_N$×^{15}N)	32	2 h	21.1 T
Sidechain ^{1}H, ^{15}N, ^{13}C chemical shift assignment and NOE					
**Sample 19**: 0.25 mM ^{15}N-, ^{13}C-labeled α7nAChR C317 TMD-ICD, 5 mM sodium acetate pH4.7, 44 mM (1%) LDAO, 25 mM NaCl; temperature: 318 K					
2D ^{1}H-^{13}C HSQC	11×64 ppm (^{1}H×^{13}C)	2048×320 (^{1}H×^{13}C)	16	1.5 h	16.4 T
3D ^{13}C-edited NOESY-HSQC	11×64×11 ppm (^{1}H×^{13}C×^{1}H)	1024×64×176 (^{1}H×^{13}C×^{1}H)	16	2 d 16 h	16.4 T (NOE mixing time: 200 ms)
**Sample 20**: 0.35 mM ^{15}N-labeled α7nAChR C317 TMD-ICD, 5 mM sodium acetate pH4.7, 66 mM (1.5%) LDAO, 25 mM NaCl; temperature: 318 K					
2D ^{1}H-^{15}N TROSY-HSQC	13×23 ppm ($^{1}H_N$×^{15}N)	1024×176 ($^{1}H_N$×^{15}N)	32	2 h	21.1 T
3D ^{15}N-edited NOESY-TROSY-HSQC	13×23×13 ppm ($^{1}H_N$×^{15}N×$^{1}H_N$)	1024×40×144 ($^{1}H_N$×^{15}N×$^{1}H_N$)	32	2 d 17 h	21.1 T (NOE mixing time: 200 ms)
Paramagnetic Relaxation Enhancement (PRE) NMR experiments					
**Sample 21**: 0.22 mM ^{15}N-labeled, V311C-MTSL-labeled α7nAChR TMD-ICD, 5 mM sodium acetate pH4.7, 44 mM (1%) LDAO, 25 mM NaCl; magnetic field: 21.1 T; temperature: 318 K					
2D ^{1}H-^{15}N TROSY-HSQC	13×23 ppm ($^{1}H_N$×^{15}N)	1024×200 ($^{1}H_N$×^{15}N)	72	4.5 h	0 and 2.2 mM ascorbic acid
**Sample 22**: 0.22 mM ^{15}N-labeled, C317-MTSL-labeled α7nAChR TMD-ICD, 5 mM sodium acetate pH4.7, 44 mM (1%) LDAO, 25 mM NaCl; magnetic field: 18.8 T; temperature: 318 K					
2D ^{1}H-^{15}N TROSY-HSQC	15×28 ppm ($^{1}H_N$×^{15}N)	1024×200 ($^{1}H_N$×^{15}N)	128	8 h	0 and 2.2 mM ascorbic acid
**Sample 22' (replicate of Sample 22)**: 0.25 mM ^{15}N-labeled, C317-MTSL-labeled α7nAChR TMD-ICD, 5 mM sodium acetate pH4.7, 44 mM (1%) LDAO, 25 mM NaCl; magnetic field: 16.4 T; temperature: 318 K					

2D ^{1}H-^{15}N TROSY-HSQC	13×23 ppm ($^{1}H_{N}$×^{15}N)	1024×176 ($^{1}H_{N}$×^{15}N)	48	3 h	0 and 2.5 mM ascorbic acid
**Sample 23**: 0.3 mM ^{15}N-labeled, C335-MTSL-labeled α7nAChR TMD-ICD, 5 mM sodium acetate pH4.7, 53 mM (1.2%) LDAO, 25 mM NaCl; magnetic field: 18.8 T; temperature: 318 K					
2D ^{1}H-^{15}N TROSY-HSQC	13×23 ppm ($^{1}H_{N}$×^{15}N)	1024×200 ($^{1}H_{N}$×^{15}N)	72	4.5 h	0 and 3.0 mM ascorbic acid
**Sample 24**: 0.3 mM ^{15}N-labeled, C342-MTSL-labeled α7nAChR TMD-ICD, 5 mM sodium acetate pH4.7, 66 mM (1.5%) LDAO, 25 mM NaCl; magnetic field: 21.1 T; temperature: 318 K					
2D ^{1}H-^{15}N TROSY-HSQC	13×23 ppm ($^{1}H_{N}$×^{15}N)	1024×200 ($^{1}H_{N}$×^{15}N)	72	4.5 h	0 and 3.0 mM ascorbic acid
**Sample 25**: 0.3 mM ^{15}N-labeled, S350C-MTSL-labeled α7nAChR TMD-ICD, 5 mM sodium acetate pH4.7, 53 mM (1.2%) LDAO, 25 mM NaCl; magnetic field: 21.1 T; temperature: 318 K					
2D ^{1}H-^{15}N TROSY-HSQC	13×23 ppm ($^{1}H_{N}$×^{15}N)	1024×200 ($^{1}H_{N}$×^{15}N)	72	4.5 h	0 and 3.0 mM ascorbic acid
**Sample 26**: 0.25 mM ^{15}N-labeled, S358C-MTSL-labeled α7nAChR TMD-ICD, 5 mM sodium acetate pH4.7, 44 mM (1%) LDAO, 25 mM NaCl; magnetic field: 21.1 T; temperature: 318 K					
2D ^{1}H-^{15}N TROSY-HSQC	13×23 ppm ($^{1}H_{N}$×^{15}N)	1024×200 ($^{1}H_{N}$×^{15}N)	96	6 h	0 and 2.5 mM ascorbic acid
**Sample 27**: 0.25 mM ^{15}N-labeled, C375-MTSL-labeled α7nAChR TMD-ICD, 5 mM sodium acetate pH4.7, 66 mM (1.5%) LDAO, 20 mM NaCl; magnetic field: 18.8 T; temperature: 318 K					
2D ^{1}H-^{15}N TROSY-HSQC	13×23 ppm ($^{1}H_{N}$×^{15}N)	1024×200 ($^{1}H_{N}$×^{15}N)	72	4.5 h	0 and 2.5 mM ascorbic acid
**Sample 28**: 0.22 mM ^{15}N-labeled, C390-MTSL-labeled α7nAChR TMD-ICD, 5 mM sodium acetate pH4.7, 53 mM (1.2%) LDAO, 25 mM NaCl; magnetic field: 18.8 T; temperature: 318 K					
2D ^{1}H-^{15}N TROSY-HSQC	13×23 ppm ($^{1}H_{N}$×^{15}N)	1024×176 ($^{1}H_{N}$×^{15}N)	96	5.5 h	0 and 2.2 mM ascorbic acid
**Sample 29**: 0.25 mM ^{15}N-labeled, L415C-MTSL-labeled α7nAChR TMD-ICD, 5 mM sodium acetate pH4.7, 44 mM (1%) LDAO, 25 mM NaCl; magnetic field: 18.8 T; temperature: 318 K					
2D ^{1}H-^{15}N TROSY-HSQC	13×23 ppm ($^{1}H_{N}$×^{15}N)	1024×200 ($^{1}H_{N}$×^{15}N)	96	6 h	0 and 2.5 mM ascorbic acid
**Sample 30**: 0.25 mM ^{15}N-labeled, C427-MTSL-labeled α7nAChR TMD-ICD, 5 mM sodium acetate pH4.7, 44 mM (1%) LDAO, 25 mM NaCl; magnetic field: 21.1 T; temperature: 318 K					
2D ^{1}H-^{15}N TROSY-HSQC	13×23 ppm ($^{1}H_{N}$×^{15}N)	1024×200 ($^{1}H_{N}$×^{15}N)	96	6 h	0 and 2.5 mM ascorbic acid
**Sample 31**: 0.25 mM ^{15}N-labeled, C435-MTSL-labeled α7nAChR TMD-ICD, 5 mM sodium acetate pH4.7, 44 mM (1%) LDAO, 25 mM NaCl; magnetic field: 18.8 T; temperature: 318 K					
2D ^{1}H-^{15}N TROSY-HSQC	13×23 ppm ($^{1}H_{N}$×^{15}N)	1024×192 ($^{1}H_{N}$×^{15}N)	72	4.5 h	0 and 2.5 mM ascorbic acid
Inter-Subunit Paramagnetic Relaxation Enhancement (PRE) NMR experiments					

**Sample 15 (inter)**: 0.24 mM C427-MTSL-labeled α7nAChR TMD-ICD mixed with 0.06 mM ^{15}N-labeled α7nAChR C427 TMD-ICD, 5 mM sodium acetate pH4.7, 44 mM (1%) LDAO, 25 mM NaCl; magnetic field: 21.1 T; temperature: 298 and 318 K					
2D ^{1}H-^{15}N TROSY-HSQC	13×23 ppm ($^{1}H_{N}$×^{15}N)	2048×192 ($^{1}H_{N}$×^{15}N)	40	2.5 h	298 K, 0 and 2.4 mM ascorbic acid
2D ^{1}H-^{15}N TROSY-HSQC	13×23 ppm ($^{1}H_{N}$×^{15}N)	2048×192 ($^{1}H_{N}$×^{15}N)	40	2.5 h	318 K, 0 and 2.4 mM ascorbic acid
**Sample 16 (control, intra)**: 0.24 mM unlabeled α7nAChR C427 TMD-ICD mixed with 0.06 mM ^{15}N-labeled, C427-MTSL-labeled α7nAChR TMD-ICD, 5 mM sodium acetate pH4.7, 44 mM (1%) LDAO, 25 mM NaCl; magnetic field: 21.1 T; temperature: 298 and 318 K					
2D ^{1}H-^{15}N TROSY-HSQC	13×23 ppm ($^{1}H_{N}$×^{15}N)	2048×192 ($^{1}H_{N}$×^{15}N)	40	2.5 h	298 K, 0 and 1.2 mM ascorbic acid
2D ^{1}H-^{15}N TROSY-HSQC	13×23 ppm ($^{1}H_{N}$×^{15}N)	2048×192 ($^{1}H_{N}$×^{15}N)	40	2.5 h	318 K, 0 and 1.2 mM ascorbic acid
**Sample 17 (inter)**: 0.28 mM V311C-MTSL-labeled α7nAChR TMD-ICD mixed with 0.07 mM ^{15}N-labeled α7nAChR V311C TMD-ICD, 5 mM sodium acetate pH4.7, 44 mM (1%) LDAO, 25 mM NaCl; magnetic field: 18.8 T; temperature: 298 and 318 K					
2D ^{1}H-^{15}N TROSY-HSQC	13×23 ppm ($^{1}H_{N}$×^{15}N)	2048×192 ($^{1}H_{N}$×^{15}N)	40	2.5 h	298 K, 0 and 2.8 mM ascorbic acid
2D ^{1}H-^{15}N TROSY-HSQC	13×23 ppm ($^{1}H_{N}$×^{15}N)	2048×192 ($^{1}H_{N}$×^{15}N)	40	2.5 h	318 K, 0 and 2.8 mM ascorbic acid
**Sample 18 (control, intra)**: 0.28 mM unlabeled α7nAChR V311C TMD-ICD mixed with 0.07 mM ^{15}N-labeled, V311C-MTSL-labeled α7nAChR TMD-ICD, 5 mM sodium acetate pH4.7, 44 mM (1%) LDAO, 25 mM NaCl; magnetic field: 18.8 T; temperature: 298 and 318 K					
2D ^{1}H-^{15}N TROSY-HSQC	13×23 ppm ($^{1}H_{N}$×^{15}N)	2048×192 ($^{1}H_{N}$×^{15}N)	40	2.5 h	298 K, 0 and 1.4 mM ascorbic acid
2D ^{1}H-^{15}N TROSY-HSQC	13×23 ppm ($^{1}H_{N}$×^{15}N)	2048×192 ($^{1}H_{N}$×^{15}N)	40	2.5 h	318 K, 0 and 1.4 mM ascorbic acid
Residual Dipolar Coupling (RDC) NMR experiments					
**Sample 19 (control)**: 0.25 mM ^{15}N-labeled, C317-labeled with a diamagnetic tag N-[S-(2-Pyridylthio)cysteaminyl]EDTA-Lu3+ α7nAChR TMD-ICD, 5 mM sodium acetate pH4.7, 66 mM (1.5%) LDAO, 25 mM NaCl; magnetic field: 21.1 T; temperature: 308 K					
2D ^{1}H-^{15}N TROSY-HSQC	16×23 ppm ($^{1}H_{N}$×^{15}N)	2048×200 ($^{1}H_{N}$×^{15}N)	64	4 h	21.1 T
**Sample 20**: 0.25 mM ^{15}N-labeled, C317-labeled with a paramagnetic tag N-[S-(2-Pyridylthio)cysteaminyl]EDTA-Tm3+ α7nAChR TMD-ICD, 5 mM sodium acetate pH4.7, 66 mM (1.5%) LDAO, 25 mM NaCl; magnetic field: 21.1 T; temperature: 308 K					
2D ^{1}H-^{15}N TROSY-HSQC	16×23 ppm ($^{1}H_{N}$×^{15}N)	2048×200 ($^{1}H_{N}$×^{15}N)	64	4 h	21.1 T

2D ^{1}H-^{15}N IPAP	16×23 ppm ($^{1}H_{N}$×^{15}N)	2048×480 ($^{1}H_{N}$×^{15}N)	80	12 h	TROSY /antiTROSY
**Sample 21**: 0.25 mM ^{15}N-labeled, C317-labeled with a paramagnetic tag N-[S-(2-Pyridylthio)cysteaminyl]EDTA-Dy3+ α7nAChR TMD-ICD, 5 mM sodium acetate pH4.7, 53 mM (1.2%) LDAO, 25 mM NaCl; magnetic field: 21.1 T; temperature: 308 K					
2D ^{1}H-^{15}N TROSY-HSQC	16×23 ppm ($^{1}H_{N}$×^{15}N)	2048×200 ($^{1}H_{N}$×^{15}N)	64	4 h	21.1 T
2D ^{1}H-^{15}N IPAP	16×23 ppm ($^{1}H_{N}$×^{15}N)	2048×480 ($^{1}H_{N}$×^{15}N)	80	12 h	TROSY /antiTROSY
**Sample 22 (control)**: 0.25 mM ^{15}N-labeled, C427-labeled with a diamagnetic tag N-[S-(2-Pyridylthio)cysteaminyl]EDTA-Lu3+ α7nAChR TMD-ICD, 5 mM sodium acetate pH4.7, 53 mM (1.2%) LDAO, 25 mM NaCl; magnetic field: 21.1 T; temperature: 308 K					
2D ^{1}H-^{15}N TROSY-HSQC	16×23 ppm ($^{1}H_{N}$×^{15}N)	2048×200 ($^{1}H_{N}$×^{15}N)	48	3 h	21.1 T
**Sample 23**: 0.20 mM ^{15}N-labeled, C427-labeled with a paramagnetic tag N-[S-(2-Pyridylthio)cysteaminyl]EDTA-Tm3+ α7nAChR TMD-ICD, 5 mM sodium acetate pH4.7, 66 mM (1.5%) LDAO, 25 mM NaCl; magnetic field: 21.1 T; temperature: 308 K					
2D ^{1}H-^{15}N TROSY-HSQC	16×23 ppm ($^{1}H_{N}$×^{15}N)	2048×200 ($^{1}H_{N}$×^{15}N)	64	4 h	21.1 T
2D ^{1}H-^{15}N IPAP	16×23 ppm ($^{1}H_{N}$×^{15}N)	2048×480 ($^{1}H_{N}$×^{15}N)	80	12 h	TROSY /antiTROSY
**Sample 24**: 0.20 mM ^{15}N-labeled, C427-labeled with a paramagnetic tag N-[S-(2-Pyridylthio)cysteaminyl]EDTA-Dy3+ α7nAChR TMD-ICD, 5 mM sodium acetate pH4.7, 66 mM (1.5%) LDAO, 25 mM NaCl; magnetic field: 21.1 T; temperature: 308 K					
2D ^{1}H-^{15}N TROSY-HSQC	16×23 ppm ($^{1}H_{N}$×^{15}N)	2048×200 ($^{1}H_{N}$×^{15}N)	64	4 h	21.1 T
2D ^{1}H-^{15}N IPAP	16×23 ppm ($^{1}H_{N}$×^{15}N)	2048×480 ($^{1}H_{N}$×^{15}N)	80	12 h	TROSY /antiTROSY
**Sample 25 (control)**: 0.24 mM ^{15}N-labeled, C219-labeled with a diamagnetic tag N-[S-(2-Pyridylthio)cysteaminyl]EDTA-Lu3+ α7nAChR TMD-ICD, 5 mM sodium acetate pH4.7, 44 mM (1.0%) LDAO, 25 mM NaCl; magnetic field: 21.1 T; temperature: 308 K					
2D ^{1}H-^{15}N TROSY-HSQC	16×23 ppm ($^{1}H_{N}$×^{15}N)	2048×200 ($^{1}H_{N}$×^{15}N)	64	4 h	21.1 T
**Sample 26**: 0.22 mM ^{15}N-labeled, C219-labeled with a paramagnetic tag N-[S-(2-Pyridylthio)cysteaminyl]EDTA-Tm3+ α7nAChR TMD-ICD, 5 mM sodium acetate pH4.7, 53 mM (1.2%) LDAO, 25 mM NaCl; magnetic field: 21.1 T; temperature: 308 K					
2D ^{1}H-^{15}N TROSY-HSQC	16×23 ppm ($^{1}H_{N}$×^{15}N)	2048×200 ($^{1}H_{N}$×^{15}N)	64	4 h	21.1 T
2D ^{1}H-^{15}N IPAP	16×23 ppm ($^{1}H_{N}$×^{15}N)	2048×480 ($^{1}H_{N}$×^{15}N)	80	12 h	TROSY /antiTROSY
^{15}N backbone dynamics					
**Sample 27-29**: 0.24 mM ^{15}N-labeled α7nAChR C317 TMD-ICD, 5 mM sodium acetate pH4.7, 66 mM (1.5%) LDAO, 25 mM NaCl; magnetic field: 16.4 T; temperature: 318 K					
2D ^{1}H-^{15}N HSQC-T_1	13×23 ppm ($^{1}H_{N}$×^{15}N)	1024×176 ($^{1}H_{N}$×^{15}N)	64	1 d 6 h	Relaxation delays: 20, 500, 1500 ms

2D ^{1}H-^{15}N HSQC-T_2	13×23 ppm (^1H_N×^{15}N)	1024×176 (^1H_N×^{15}N)	72	3 d	Relaxation delays: 16, 32, 112, 160, 600 ms
2D ^{1}H-^{15}N HSQC-hetNOE	13×23 ppm (^1H_N×^{15}N)	1024×352 (^1H_N×^{15}N)	80	2 d 18 h	16.4 T
α7nAChR TMD-ICD samples in nanodiscs and bicelles					
**Sample 30 (nanodiscs)**: 0.20 mM ^{15}N-labeled α7nAChR C317 TMD-ICD, 5 mM sodium acetate pH4.8, 10 mM asolectin, 0.25 mM MSP1D1, 25 mM NaCl; magnetic field: 18.8 T; temperature: 318 K					
2D ^{1}H-^{15}N TROSY-HSQC	13×23 ppm (^1H_N×^{15}N)	1024×200 (^1H_N×^{15}N)	64	4 h	18.8 T
**Sample 31 (nanodiscs)**: 0.20 mM ^{15}N-labeled α7nAChR C317 TMD-ICD, 5 mM sodium acetate pH4.8, 10 mM DMPC, 0.25 mM MSP1D1, 25 mM NaCl; magnetic field: 21.1 T; temperature: 308 K					
2D ^{1}H-^{15}N TROSY-HSQC	13×23 ppm (^1H_N×^{15}N)	1024×176 (^1H_N×^{15}N)	64	4 h	21.1 T
**Sample 32 (nanodiscs)**: 0.18 mM ^{15}N-labeled α7nAChR C317 TMD-ICD, 5 mM sodium acetate pH4.8, 10 mM DMPC, 0.25 mM MSP1D1, 25 mM NaCl; magnetic field: 21.1 T; temperature: 308 K					
2D ^{1}H-^{15}N TROSY-HSQC	13×23 ppm (^1H_N×^{15}N)	1024×176 (^1H_N×^{15}N)	64	4 h	21.1 T
**Sample 33 (bicelles)**: 0.15 mM ^{15}N-labeled α7nAChR C317 TMD-ICD, 5 mM sodium acetate pH4.9, 10 mM DMPC, 20 mM DHPC, 25 mM NaCl; magnetic field: 18.8 T; temperature: 318 K					
2D ^{1}H-^{15}N TROSY-HSQC	13×23 ppm (^1H_N×^{15}N)	1024×176 (^1H_N×^{15}N)	64	4 h	18.8 T
Paramagnetic Relaxation Enhancement (PRE) NMR experiments of **α7nAChR TMD-ICD samples in nanodiscs and bicelles**					
**Sample 34 (nanodiscs)**: 0.20 mM ^{15}N-labeled, C317-MTSL-labeled α7nAChR TMD-ICD, 5 mM sodium acetate pH4.9, 10 mM DMPC, 0.25 mM MSP1D1, 25 mM NaCl; magnetic field: 21.1 T; temperature: 308 K					
2D ^{1}H-^{15}N TROSY-HSQC	13×27 ppm (^1H_N×^{15}N)	1024×176 (^1H_N×^{15}N)	112	7 h	0 and 2.0 mM ascorbic acid
**Sample 35 (bicelles)**: 0.10 mM ^{15}N-labeled, V311C-MTSL-labeled α7nAChR TMD-ICD, 5 mM sodium acetate pH5.0, 15 mM DMPC, 30 mM DHPC, 25 mM NaCl; magnetic field: 21.1 T; temperature: 308 K					
2D ^{1}H-^{15}N TROSY-HSQC	13×23 ppm (^1H_N×^{15}N)	1024×176 (^1H_N×^{15}N)	128	8 h	0 and 2.0 mM ascorbic acid

*A recycle delay (D1) of 1 s was used in all NMR experiments, except 2D relaxation R_1, R_2 and ^{1}H-^{15}N heteronuclear NOE (3 s) and a BEST (band-selective excitation short-transient) version of 3D HNCA, and HNCO (0.4 s).

All samples contain 20 μM DSS for the reference of the 0 ppm ^{1}H chemical shift.

Supplementary References

1. Bondarenko V, Mowrey DD, Tillman TS, Seyoum E, Xu Y, Tang P. NMR structures of the human alpha7 nAChR transmembrane domain and associated anesthetic binding sites. *Biochim Biophys Acta* **1838**, 1389-1395 (2014).

2. Dalmas O, Hyde HC, Hulse RE, Perozo E. Symmetry-constrained analysis of pulsed double electron-electron resonance (DEER) spectroscopy reveals the dynamic nature of the KcsA activation gate. *J Am Chem Soc* **134**, 16360-16369 (2012).

3. Pannier M, Veit S, Godt A, Jeschke G, Spiess HW. Dead-time free measurement of dipole-dipole interactions between electron spins. *J Magn Reson* **142**, 331-340 (2000).

4. Hurst RS*, et al*. A novel positive allosteric modulator of the alpha7 neuronal nicotinic acetylcholine receptor: in vitro and in vivo characterization. *J Neurosci* **25**, 4396-4405 (2005).

5. Gill JK, Savolainen M, Young GT, Zwart R, Sher E, Millar NS. Agonist activation of alpha7 nicotinic acetylcholine receptors via an allosteric transmembrane site. *Proceedings of the National Academy of Sciences of the United States of America* **108**, 5867-5872 (2011).

6. Basak S*, et al*. Cryo-EM structure of 5-HT3A receptor in its resting conformation. *Nat Commun* **9**, 514 (2018).

7. Hassaine G*, et al*. X-ray structure of the mouse serotonin 5-HT3 receptor. *Nature* **512**, 276-281 (2014).

8. Song Y*, et al*. High-resolution comparative modeling with RosettaCM. *Structure* **21**, 1735-1742 (2013).

9. Tyka MD*, et al*. Alternate states of proteins revealed by detailed energy landscape mapping. *J Mol Biol* **405**, 607-618 (2011).

10. Nivon LG, Moretti R, Baker D. A Pareto-optimal refinement method for protein design scaffolds. *PLoS One* **8**, e59004 (2013).

11. Conway P, Tyka MD, DiMaio F, Konerding DE, Baker D. Relaxation of backbone bond geometry improves protein energy landscape modeling. *Protein Sci* **23**, 47-55 (2014).

12. Pordes R*, et al*. The open science grid. In: *J. Phys. Conf. Ser.* (ed^(eds) (2007).

13. Sfiligoi I, Bradley DC, Holzman B, Mhashilkar P, Padhi S, Wurthwein F. The pilot way to grid resources using glideinWMS. In: *Computer Science and Information Engineering, 2009 WRI World Congress on* (ed^(eds). IEEE (2009).

14. Ramachandran S, Kota P, Ding F, Dokholyan NV. Automated minimization of steric clashes in protein structures. *Proteins* **79**, 261-270 (2011).

15. Adams PD*, et al*. PHENIX: a comprehensive Python-based system for macromolecular structure solution. *Acta Crystallogr D Biol Crystallogr* **66**, 213-221 (2010).

16. Iwahara J, Anderson DE, Murphy EC, Clore GM. EDTA-derivatized deoxythymidine as a tool for rapid determination of protein binding polarity to DNA by intermolecular paramagnetic relaxation enhancement. *J Am Chem Soc* **125**, 6634-6635 (2003).

17. Iwahara J, Schwieters CD, Clore GM. Ensemble approach for NMR structure refinement against (1)H paramagnetic relaxation enhancement data arising from a flexible paramagnetic group attached to a macromolecule. *J Am Chem Soc* **126**, 5879-5896 (2004).

18. Clore GM, Tang C, Iwahara J. Elucidating transient macromolecular interactions using paramagnetic relaxation enhancement. *Curr Opin Struct Biol* **17**, 603-616 (2007).

19. Wishart DS, Sykes BD. The 13C chemical-shift index: a simple method for the identification of protein secondary structure using 13C chemical-shift data. *J Biomol NMR* **4**, 171-180 (1994).

20. Shen Y*, et al*. Consistent blind protein structure generation from NMR chemical shift data. *Proceedings of the National Academy of Sciences of the United States of America* **105**, 4685-4690 (2008).

21. Shen Y, Delaglio F, Cornilescu G, Bax A. TALOS+: a hybrid method for predicting protein backbone torsion angles from NMR chemical shifts. *J Biomol NMR* **44**, 213-223 (2009).

22. Kamen DE, Cahill SM, Girvin ME. Multiple alignment of membrane proteins for measuring residual dipolar couplings using lanthanide ions bound to a small metal chelator. *J Am Chem Soc* **129**, 1846-1847 (2007).

23. Ikegami T*, et al*. Novel techniques for weak alignment of proteins in solution using chemical tags coordinating lanthanide ions. *J Biomol NMR* **29**, 339-349 (2004).

24. Bax A. Weak alignment offers new NMR opportunities to study protein structure and dynamics. *Protein Sci* **12**, 1-16 (2003).

25. Prestegard JH, al-Hashimi HM, Tolman JR. NMR structures of biomolecules using field oriented media and residual dipolar couplings. *Q Rev Biophys* **33**, 371-424 (2000).

26. Gillespie JR, Shortle D. Characterization of long-range structure in the denatured state of staphylococcal nuclease. II. Distance restraints from paramagnetic relaxation and calculation of an ensemble of structures. *J Mol Biol* **268**, 170-184 (1997).

27. Gillespie JR, Shortle D. Characterization of long-range structure in the denatured state of staphylococcal nuclease. I. Paramagnetic relaxation enhancement by nitroxide spin labels. *J Mol Biol* **268**, 158-169 (1997).

28. Battiste JL, Wagner G. Utilization of site-directed spin labeling and high-resolution heteronuclear nuclear magnetic resonance for global fold determination of large proteins with limited nuclear overhauser effect data. *Biochemistry* **39**, 5355-5365 (2000).

29. Liang B, Bushweller JH, Tamm LK. Site-directed parallel spin-labeling and paramagnetic relaxation enhancement in structure determination of membrane proteins by solution NMR spectroscopy. *J Am Chem Soc* **128**, 4389-4397 (2006).

30. Stein RA, Beth AH, Hustedt EJ. A Straightforward Approach to the Analysis of Double Electron-Electron Resonance Data. *Methods Enzymol* **563**, 531-567 (2015).

31. Zhao Y*, et al*. Structural basis of human alpha7 nicotinic acetylcholine receptor activation. *Cell Res* **31**, 713-716 (2021).

32. Noviello CM*, et al*. Structure and gating mechanism of the alpha7 nicotinic acetylcholine receptor. *Cell*, (2021).

33. Humphrey W, Dalke A, Schulten K. VMD: visual molecular dynamics. *J Mol Graph* **14**, 33-38, 27-38 (1996).

34. Farrow NA*, et al*. Backbone dynamics of a free and phosphopeptide-complexed Src homology 2 domain studied by 15N NMR relaxation. *Biochemistry* **33**, 5984-6003 (1994).

35. Ma D*, et al*. NMR studies of a channel protein without membranes: structure and dynamics of water-solubilized KcsA. *Proceedings of the National Academy of Sciences of the United States of America* **105**, 16537-16542 (2008).

36. Kneller JM, Lu M, Bracken C. An effective method for the discrimination of motional anisotropy and chemical exchange. *J Am Chem Soc* **124**, 1852-1853 (2002).

37. Rahman MM*, et al*. Structure of the Native Muscle-type Nicotinic Receptor and Inhibition by Snake Venom Toxins. *Neuron* **106**, 952-962 e955 (2020).

38. Gharpure A*, et al*. Agonist Selectivity and Ion Permeation in the alpha3beta4 Ganglionic Nicotinic Receptor. *Neuron* **104**, 501-511 e506 (2019).

39. Basak S, Gicheru Y, Rao S, Sansom MSP, Chakrapani S. Cryo-EM reveals two distinct serotonin-bound conformations of full-length 5-HT3A receptor. *Nature* **563**, 270-274 (2018).

40. Smart OS, Neduvelil JG, Wang X, Wallace BA, Sansom MS. HOLE: a program for the analysis of the pore dimensions of ion channel structural models. *J Mol Graph* **14**, 354-360, 376 (1996).

41. Bondarenko V*, et al*. (19)F Paramagnetic Relaxation-Based NMR for Quaternary Structural Restraints of Ion Channels. *ACS Chem Biol* **14**, 2160-2165 (2019).

42. Chen VB*, et al*. MolProbity: all-atom structure validation for macromolecular crystallography. *Acta Crystallogr D Biol Crystallogr* **66**, 12-21 (2010).

43. Schwieters CD, Kuszewski JJ, Tjandra N, Clore GM. The Xplor-NIH NMR molecular structure determination package. *J Magn Reson* **160**, 65-73 (2003).

44. Madeira F*, et al*. The EMBL-EBI search and sequence analysis tools APIs in 2019. *Nucleic Acids Res* **47**, W636-W641 (2019).
