# Supplementary material for: Structures of highly flexible intracellular domain of human α7 nicotinic acetylcholine receptor
Source: Nat Commun. 2022 Feb 10;13:793. doi: 10.1038/s41467-022-28400-x (PMC8831596; doi:10.1038/s41467-022-28400-x)
Supplement: Supplementary file 3 — Reporting Summary [file 41467_2022_28400_MOESM3_ESM.pdf]

nature portfolio

Corresponding author(s): Pei Tang

Last updated by author(s): Dec 10, 2021

Reporting Summary

Nature Portfolio wishes to improve the reproducibility of the work that we publish. This form provides structure for consistency and transparency in reporting. For further information on Nature Portfolio policies, see our Editorial Policies and the Editorial Policy Checklist.

Statistics

For all statistical analyses, confirm that the following items are present in the figure legend, table legend, main text, or Methods section.

n/a	Confirmed	
☐	☒	The exact sample size (*n*) for each experimental group/condition, given as a discrete number and unit of measurement
☐	☒	A statement on whether measurements were taken from distinct samples or whether the same sample was measured repeatedly
☒	☐	The statistical test(s) used AND whether they are one- or two-sided<br>*Only common tests should be described solely by name; describe more complex techniques in the Methods section.*
☒	☐	A description of all covariates tested
☒	☐	A description of any assumptions or corrections, such as tests of normality and adjustment for multiple comparisons
☐	☒	A full description of the statistical parameters including central tendency (e.g. means) or other basic estimates (e.g. regression coefficient) AND variation (e.g. standard deviation) or associated estimates of uncertainty (e.g. confidence intervals)
☒	☐	For null hypothesis testing, the test statistic (e.g. *F*, *t*, *r*) with confidence intervals, effect sizes, degrees of freedom and *P* value noted<br>*Give P values as exact values whenever suitable.*
☒	☐	For Bayesian analysis, information on the choice of priors and Markov chain Monte Carlo settings
☒	☐	For hierarchical and complex designs, identification of the appropriate level for tests and full reporting of outcomes
☒	☐	Estimates of effect sizes (e.g. Cohen's *d*, Pearson's *r*), indicating how they were calculated

Our web collection on statistics for biologists contains articles on many of the points above.

Software and code

Policy information about availability of computer code

Data collection	Electrophysiology: Clampex 10.6 software (Molecular Devices); SPR: Biacore 3000 software 4.1.2; NMR data acquisition: Bruker TopSpin 2.1 and 3.1 - 3.5; ESR: Xepr 2.6b.176
Data analysis	1. GraphPad Prism 7.04 for nonlinear regressions and statistical analysis<br>2. NMRPipe 10.9 and Sparky 3.19 for NMR spectral process and analysis<br>3. Origin Lab Corp OriginPro 8.5 for NMR data processing<br>4. Mathworks Matlab 2020b for data processing.<br>5. DD version 7C (requires Matlab) for ESR data processing and analysis<br>6. CS-Rosetta 3.7 for generating fragment libraries; RosettaCM in Rosetta 3.7 for structure calculations; Rosetta FastRelax 3.7 for structure relaxation<br>7. Chiron: rapid protein energy minimization server (2011)- for minimizing steric clashes<br>8. Phenix version1.19 for geometry optimization and structure statistics<br>9. MolProbity server version 4.5.1-Duke University and Xplor-NIH version 3.4 for evaluating structure quality and statistics<br>10. HOLE version 2.0 for channel pore analysis<br>11. VMD version 1.9.3 for displaying and analyzing protein structures<br>12. BIAEvaluation 4.1.1 for SPR data processing<br>13. Clampfit 10.6 for electrophysiology data processing.<br>14. Clustal Omega 1.2.4 for sequence alignment

For manuscripts utilizing custom algorithms or software that are central to the research but not yet described in published literature, software must be made available to editors and reviewers. We strongly encourage code deposition in a community repository (e.g. GitHub). See the Nature Portfolio guidelines for submitting code & software for further information.

Data

Policy information about availability of data

All manuscripts must include a data availability statement. This statement should provide the following information, where applicable:

- Accession codes, unique identifiers, or web links for publicly available datasets
- A description of any restrictions on data availability
- For clinical datasets or third party data, please ensure that the statement adheres to our policy

The atomic coordinates and structural restraints for 15 structures of the apo α7nAChR TMD+ICD have been deposited in the Protein Data Bank with the accession code 7RPM [https://www.rcsb.org/structure/unreleased/7RPM]. The source data underlying Figs. 1, 2, 5 and Supplementary Figs. 2, 4, 5, 8, 10, 13, 15 are provided as a Source Data file. The chemical shift values have been deposited in the Biological Magnetic Resonance Data Bank (BMRB), accession number 30939 [https://dx.doi.org/10.13018/BMR30939]. Other data that support the findings of this study are available upon reasonable request to the corresponding author.

Field-specific reporting

Please select the one below that is the best fit for your research. If you are not sure, read the appropriate sections before making your selection.

☒ Life sciences ☐ Behavioural & social sciences ☐ Ecological, evolutionary & environmental sciences

For a reference copy of the document with all sections, see nature.com/documents/nr-reporting-summary-flat.pdf

Life sciences study design

All studies must disclose on these points even when the disclosure is negative.

Sample size	For structure determination, sample size is not applicable. Sample sizes for electrophysiology and SPR were based on conventions for these techniques.
Data exclusions	The data collected from leaky/unhealthy oocytes in electrophysiological recordings were not included in analysis. NMR data collected in DMPC/DHPC bicelles or micelles other than LDAO were not used for structural restraints because of an instability of proteins in these membrane mimetics and a worse quality of NMR spectra.
Replication	Electrophysiology experiments were performed on independent oocytes, from different surgeries. Number of replicates for experimental data are indicated in the legends.
Randomization	Randomly selected healthy oocytes harvested from different frogs were injected with TMD+ICD to establish a single experimental group.
Blinding	There was no need for blinding in this study because there was no different treatment for the subjects.

Reporting for specific materials, systems and methods

We require information from authors about some types of materials, experimental systems and methods used in many studies. Here, indicate whether each material, system or method listed is relevant to your study. If you are not sure if a list item applies to your research, read the appropriate section before selecting a response.

Materials & experimental systems

n/a	Involved in the study	
☒	☐	Antibodies
☒	☐	Eukaryotic cell lines
☒	☐	Palaeontology and archaeology
☒	☐	Animals and other organisms
☒	☐	Human research participants
☒	☐	Clinical data
☒	☐	Dual use research of concern

Methods

n/a	Involved in the study	
☒	☐	ChIP-seq
☒	☐	Flow cytometry
☒	☐	MRI-based neuroimaging
